# Dynamic Arginine Methylation of YBX1 Relay Controls Its Phase Separation and Chemoradiotherapy Resistance in Rectal Cancer

**DOI:** 10.1002/advs.202502786

**Published:** 2025-10-28

**Authors:** Yunxing Shi, Taixuan Wan, Shaoru Liu, Huashan Liu, Ziwei Zeng, Wenxin Li, Zhenxing Liang, Li Xiong, Shuanglin Luo, Yunfei Yuan, Liang Huang, Liang Kang

**Affiliations:** ^1^ Department of General Surgery (Colorectal Surgery) The Sixth Affiliated Hospital Sun Yat‐sen University Guangzhou 510000 China; ^2^ Guangdong Provincial Key Laboratory of Colorectal and Pelvic Floor Diseases The Sixth Affiliated Hospital Sun Yat‐sen University Guangzhou 510000 China; ^3^ State Key Laboratory of Oncology in South China Guangdong Provincial Clinical Research for Cancer Sun Yat‐Sen University Cancer Center Guangzhou 510030 China

**Keywords:** arginine methylation, chemoradiotherapy resistance, PRMT3, rectal cancer, YBX1

## Abstract

Although neoadjuvant chemoradiotherapy is the standard treatment and significantly improves prognosis of locally advanced rectal cancer, patients may develop primary or secondary therapy resistance. Here, through functional screening, we identify PRMT3 as a key driver of chemoradiotherapy resistance in rectal cancer. PRMT3 induces the arginine methylation of Y‐box binding protein 1 (YBX1), which suppresses YBX1's capacity for phase separation in cytoplasm and is imperative for its nucleus translocation. Subsequently, lysine‐specific demethylase 4A (KDM4A) demethylates YBX1 to unmask the R247 residue and promote its phase separation in the nucleus, which strengthens its ability to transcriptionally regulate expressions of ATP‐binding cassette (ABC) transporters and homologous recombination (HR) repair genes and reinforces chemoradiotherapy resistance. Of particular translational importance, high‐throughput FDA‐approved drugs library screening identifies acipimox as a potent PRMT3 inhibitor and chemoradiotherapy sensitizer. This study offers a tangible prospect for improving therapeutic outcomes in rectal cancer patients in a clinically relevant setting.

## Graphical abstract

Protein arginine methyltransferase 3 (PRMT3) induces the arginine methylation of Y‐box binding protein 1 (YBX1), which suppresses phase separation and is imperative for its nuclear translocation. Subsequently, lysine‐specific demethylase 4A demethylates YBX1 to unmask the R247 residue and promote its phase separation, which transcriptionally regulates expressions of targets and reinforces chemoradiotherapy resistance.

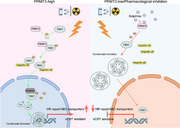

## 1 Introduction

Rectal cancer stands as the world's third most prevalent and fatal malignancy.^[^ ^1^ ^]^ Over 30% of rectal cancer have progressed to the middle or late stage at the time of diagnosis.^[^ ^2^ ^]^ Neoadjuvant chemoradiotherapy precedes surgical intervention as the gold standard for locally advanced cases.^[^ ^3^ ^]^ However, a subset of patients develops primary or secondary resistance, compromising the efficacy of such therapies and facilitating tumor recurrence and metastasis.^[^ ^4^ ^]^ The achievement of a pathological complete response (pCR) post‐neoadjuvant treatment is limited to ≈15%–27% of patients, while 20%–40% display resistance and minimal therapeutic benefit.^[^ ^4^ ^]^ Therefore, further elucidating the molecular mechanisms of rectal cancer chemoradiotherapy resistance, identifying more potential drugs to overcome chemoradiotherapy resistance, and screening for treatment‐advantaged populations have important clinical significance in improving the treatment effect of rectal cancer, and especially for patients with middle and late‐stage rectal cancer.

Multiple mechanisms, including DNA damage repair, exosome secretion, cell cycle regulation, tumor metabolism, tumor stemness, and gut microbiota, have been implicated in the de novo resistance and acquired resistance to chemoradiotherapy resistance.^[^ ^5^, ^6^, ^7^, ^8^ ^]^ Regulators of post‐translational modifications (PTM), including histone lysine methyltransferases (KMT), histone lysine demethylase (KDM), and protein arginine methyltransferases (PRMT), participate in key biological processes and have emerged as critical players in tumorigenesis and therapeutic resistance (e.g., chemotherapy, targeted therapy, and immunotherapy) in various cancer types.^[^ ^9^, ^10^, ^11^, ^12^ ^]^ Currently, there is still very rare research on the role of post‐translational modifications of proteins in the resistance to radiotherapy and chemotherapy in rectal cancer. Therefore, there is an urgent need for large‐scale screening and in‐depth studies to provide clues for clinical treatments.

Through meticulous functional genomic screening, our study has pinpointed PRMT3 as a pivotal contributor to chemoradiotherapy resistance in rectal cancer. PRMT3 orchestrates YBX1 methylation, suppressing its phase separation and fostering nucleus translocation. Subsequently, KDM4A mediates YBX1 demethylation, exposing R247 to facilitate nucleus phase separation, which bolsters transcriptional control of ABC transporter and HR repair genes, thereby fortifying chemoradiotherapy resistance. Notably, our high‐throughput screening of an FDA‐approved drug library has revealed acipimox as a robust PRMT3 inhibitor. Collectively, we define a methylation–demethylation of YBX1‐R247 relay spatiotemporally controls phase separation and chemoradiotherapy resistance. Importantly, the therapeutic potential of acipimox as a chemoradiotherapy sensitizer presents a clinically translatable strategy for overcoming resistance in rectal cancer therapy.

## 2 Results

### 2.1 PRMT3 Is a Key PTMs Mediating Neoadjuvant Chemoradiation Resistance in Rectal Cancer

**Figure 1:**
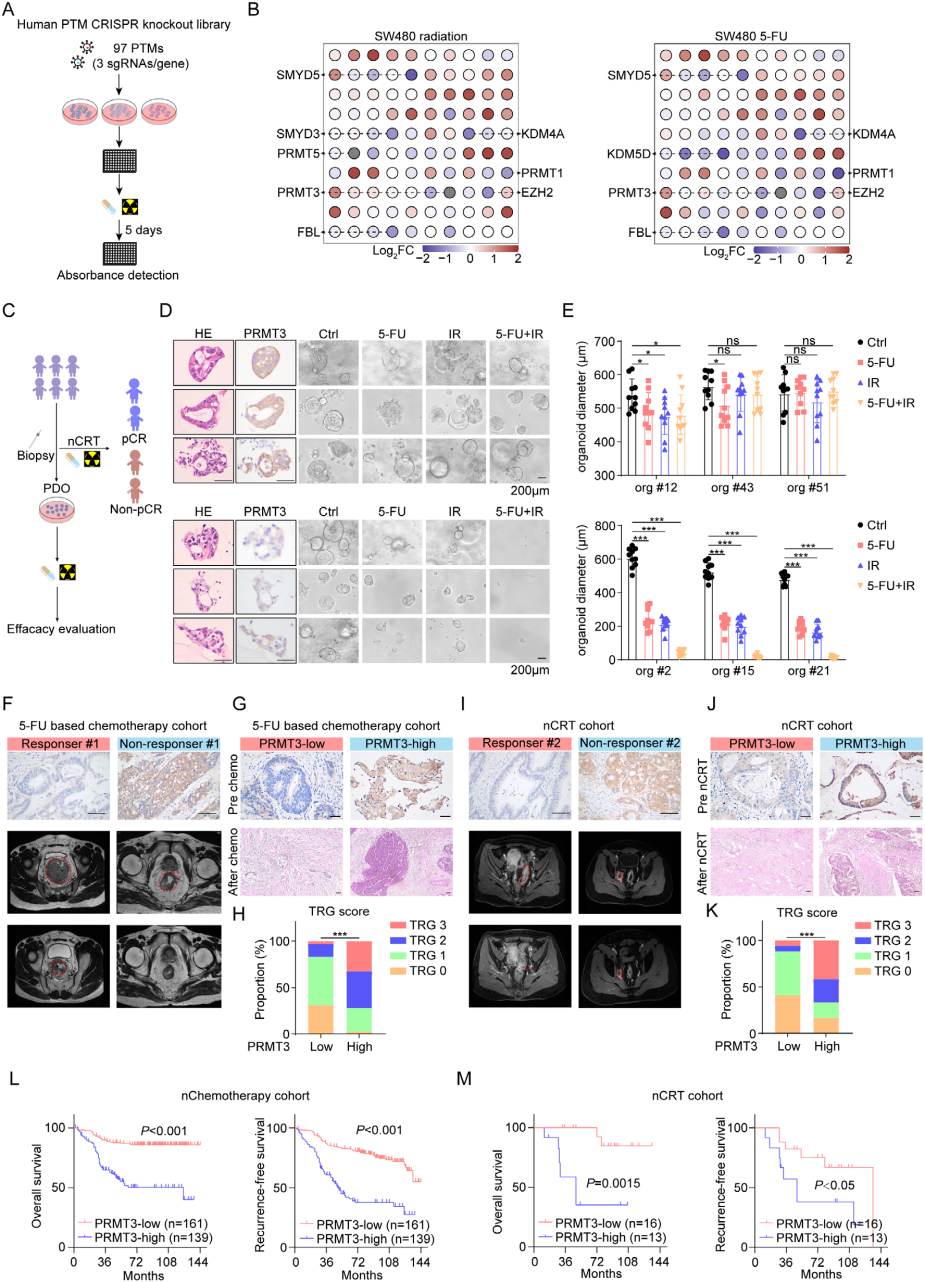
PRMT3 is a key driver of chemoradiotherapy resistance and associated with poor clinical outcomes. A) Schematic of gene‐editing coupled functional screening strategy to discover candidate protein methyltransferases (PTM) responsible for chemoradiotherapy resistance. B) The effect of PTM knockout on chemoresistance and radiotherapy resistance of SW480 cell line. Cells were treated with vehicle, 5‐FU (6 × 10^−3^ m), or radiation (8 Gy) for 5 d. The cell viability was evaluated with CCK‐8 assay. C) Schematic of PDOs culture, grouping, and efficacy evaluation. D) The association between PRMT3 expression of PDOs and chemoradiotherapy resistance. E) The baseline and post‐treatment CT images of rectal cancer patients, who had low and high PRMT3 expression, respectively, showed the patients’ response to the 5‐FU based chemotherapy. Scale bar, 50 mm. F) The post‐treatment H&E staining of rectal cancer tissues, who had low and high PRMT3 expression, respectively, showed the patients’ response to the 5‐FU based chemotherapy. Scale bar, 50 mm. G) The TRG score evaluation of rectal cancer patients, who had low and high PRMT3 expression, respectively, showed the patients’ response to the 5‐FU based chemotherapy. H) The baseline and post‐treatment CT images of rectal cancer patients, who had low and high PRMT3 expression, respectively, showed the patients’ response to the neoadjuvant chemoradiotherapy. Scale bar, 50 mm. I) The post‐treatment H&E staining of rectal cancer tissues, who had low and high PRMT3 expression, respectively, showed the patients’ response to the neoadjuvant chemoradiotherapy. Scale bar, 50 mm. J) The TRG score evaluation of rectal cancer patients, who had low and high PRMT3 expression, respectively, showed the patients’ response to the neoadjuvant chemoradiotherapy. K) Kaplan–Meier overall survival and disease‐free survival curves of individuals with different PRMT3 expression in the 5‐FU based chemotherapy cohort, PRMT3‐high, *n* = 139; PRMT3‐low, *n* = 161. L) Kaplan–Meier overall survival and disease‐free survival curves of individuals with different PRMT3 expression in the neoadjuvant chemoradiotherapy cohort, PRMT3‐high, *n* = 13; PRMT3‐low, *n* = 16.

To identify the key PTM enzymes involved in chemoradiation resistance in rectal cancer, we selected the SW480 cell line due to its high resistance to 5‐FU and radiation. We established a comprehensive panel of individual knockout SW480 cell lines for 97 characterized and putative PTM enzymes. The cell lines were then treated with chemoradiation (**Figure** **1**A; Figure S1A,B and Table S1, Supporting Information). These cell lines were exposed to a therapeutically effective dose of a cytotoxic agent that significantly inhibited the proliferation of SW480 cells (Figure S1C, Supporting Information). As shown in Figure 1B, majority of PTMs conferred chemoradiation resistance in rectal cancer, as indicated with a decrease cell viability when indicated genes were knocked out. Among these PTMs exhibiting consistent effects on chemoradiation resistance (Figure 1C), several oncogenic modifiers, including SMYD3, PRMT3, PRMT5, and FBL, were noted, which have been previously implicated in multidrug resistance across various cancers.^[^ ^11^, ^13^, ^14^ ^]^ Given our prior demonstration of PRMT3's central role in oxaliplatin resistance and immunotherapy evasion in hepatocellular carcinoma,^[^ ^13^ ^]^ we elected to concentrate our investigation on PRMT3. Consistent with our screening result, the expression level of PRMT3 in CRC cell lines were notably correlated with their survival ability under chemoradiation treatment (Figure S1D, Supporting Information).

In pursuit of validating PRMT3's involvement in neoadjuvant chemoradiation resistance in rectal cancer, we generated several rectal cancer patient‐derived organoid lines (PDO) which develops a dense 3D morphology (Figure 1D). Upon exposure to IR, 5‐FU, or their combined therapy, PDOs expressing higher levels of PRMT3 demonstrated heightened proliferative capacity and resistance, manifesting as larger organoids compared to those with lower PRMT3 expression (Figure 1D,E). To verify whether PRMT3 expression could predict the efficacy of neoadjuvant chemoradiation, we enrolled 300 patients diagnosed with advanced colorectal cancer in cohort 1. The tissue samples were obtained from colorectal cancer patients who undergo chemotherapy through endoscopy biopsy and surgical resection. Strong and moderate IHC staining intensity of PRMT3 was defined as PRMT3‐high whereas weak and negative staining was defined as PRMT3‐low (Figure 1F,G). Our analysis revealed that patients exhibiting favorable responses to chemotherapy, as assessed by computed tomography (CT) imaging and hematoxylin and eosin (H&E) staining, had lower PRMT3 expression levels in comparison to those with high PRMT3 expression. Utilizing the Tumor Regression Grade (TRG) scoring system (Figure 1F,G). Based on the TRG score,^[^ ^15^ ^]^ we observed a significantly higher proportion of PRMT3‐Low patients achieving TRG 0 and TRG 1 (82.7%), contrasting with PRMT3‐High patients, where 71.79% experienced TRG 2 and TRG 3 (Figure 1H). An independent validation in Cohort 2, comprising 29 patients who received neoadjuvant chemoradiation therapy, echoed these findings. Lower PRMT3 expression correlated with enhanced responsiveness to therapy, as confirmed by CT, H&E staining, and TRG assessment (Figure 1I–K). Of utmost clinical relevance, elevated PRMT3 expression was significantly correlated with shortened overall survival following both chemotherapy and chemoradiation therapy (Figure 1L), with similar trends observed in another study focusing on neoadjuvant chemotherapy outcomes (Figure 1M; Figure S1E,F, Supporting Information). Collectively, these data robustly implicate high PRMT3 expression as a pivotal PTM driving chemoradiation resistance in advanced colorectal cancer, associating it with inferior prognosis and attenuated responsiveness to neoadjuvant chemoradiation regimens.

### 2.2 PRMT3 Confers Chemoradiation Resistance in Rectal Cancer

**Figure 2:**
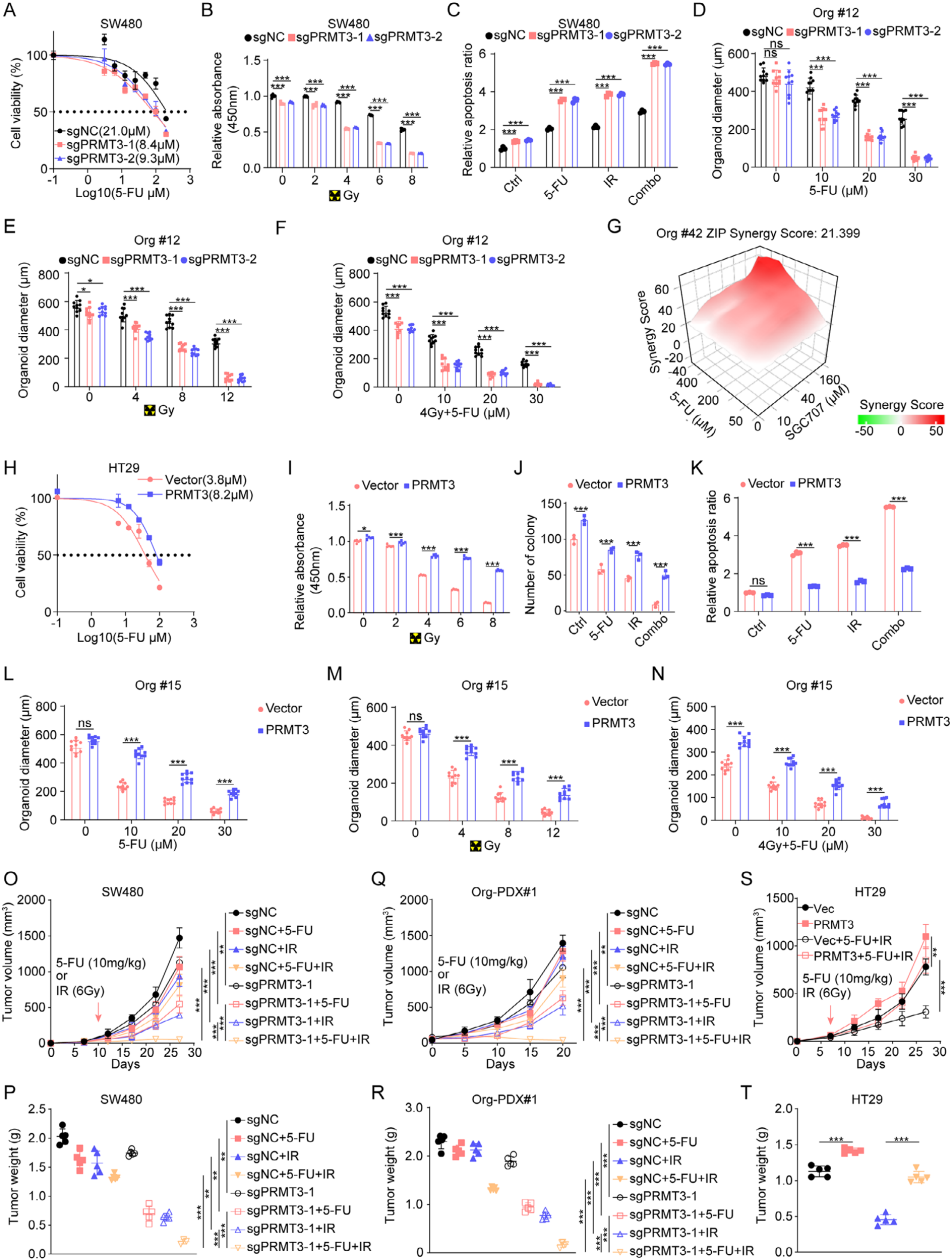
PRMT3 promotes chemoradiotherapy resistance in rectal cancer. A) The IC50 of 5‐FU in *PRMT3*‐KO and control SW480 cells. B) Cell viability of *PRMT3*‐KO and control SW480 cells after irradiation. C) Apoptosis ratio of *PRMT3*‐KO and control SW480 cells after treated with 5‐FU, irradiation and combination. D) Diameter of *PRMT3*‐KO and control PDO#12 after treated with 5‐FU. E) Diameter of *PRMT3*‐KO and control PDO#12 after irradiation. F) Diameter of *PRMT3*‐KO and control PDO#12 after treated with 5‐FU and irradiation (4 Gy). G) Synergy score of PDO#12 treated with SGC707 and 5‐FU. H) The IC50 of 5‐FU in *PRMT3*‐OE and control HT29 cells. I) Cell viability of *PRMT3*‐OE and control HT29 cells after irradiation. J) Colony number of *PRMT3*‐OE and control HT29 cells after treated with 5‐FU, irradiation and combination. K) Apoptosis ratio of *PRMT3*‐OE and control HT29 cells after treated with 5‐FU, irradiation and combination. L) Diameter of *PRMT3*‐OE and control PDO#15 after treated with 5‐FU. M) Diameter of *PRMT3*‐OE and control PDO#15 after irradiation. N) Diameter of *PRMT3*‐OE and control PDO#15 after treated with 5‐FU and irradiation (4 Gy). The measurement of O) tumor volumes and P) tumor weights of *PRMT3*‐KO SW480 cells and control cells treated with 5‐FU, irradiation and combination. The measurement of Q) tumor volumes and R) tumor weights of *PRMT3*‐KO and control PDO#12 treated with 5‐FU, irradiation and combination. The measurement of S) tumor volumes and T) tumor weights of *PRMT3*‐OE and control HT29 cells treated with 5‐FU and irradiation.

To investigate the direct impact of PRMT3 on inducing chemoradiation resistance in colorectal cancer, we generated *PRMT3*‐KO SW480 and SW620 cell lines and the efficiency of *PRMT3*‐KO by two independent sgRNAs in the pooled cells was confirmed by Western blot (Figure S2A, Supporting Information). Our findings revealed that ablation of PRMT3 significantly decreased the half‐maximal inhibitory concentration (IC50) of 5‐FU in both SW480 and SW620 cell lines (**Figure** **2**A; Figure S2B, Supporting Information). In addition, *PRMT3*‐KO cells exhibited an augmented sensitivity to the growth‐suppressive effects of 5‐FU and radiation, as evidenced by Cell Counting Kit‐8 (CCK‐8) viability assays and colony formation assays (Figure 2B; Figure S2C,D, Supporting Information). Furthermore, *PRMT3*‐KO sensitize these cells to 5‐FU and radiation‐induced apoptosis compared to CTRL cells as shown by FACS analysis of Annexin V staining (Figure 2C). In parallel, we generated *PRMT3*‐KO patient‐derived organoids (PDOs) utilizing two independent sgRNAs, which displayed diminished growth and resistance characteristics relative to CTRL PDOs, as indicated by their reduced organoid dimensions (Figure 2E,F). Given that 5‐FU is just one of the drugs used in neoadjuvant chemotherapy for rectal cancer, we explored whether PRMT3 promoted resistance of rectal cancer to other commonly used drugs. Results showed that *PRMT3*‐KO also enhanced the sensitivity of rectal cancer to oxaliplatin and irinotecan (Figure S2E,F, Supporting Information). To further access whether the effects of PRMT3 on chemoradiotherapy resistance were dependent on its enzyme activity, we re‐expressed the WT‐PRMT3 and enzyme dead PRMT3 in *PRMT3*‐KO SW480 cells. As expected, WT‐PRMT3 re‐expressing completely rescued the effects of *PRMT3*‐KO on chemoradiotherapy resistance. However, enzyme dead PRMT3 re‐expressing failed to rescue the effects of *PRMT3*‐KO on chemoradiotherapy resistance of SW480 cells, as reflected by the IC50 and CCK‐8 assay (Figure S2G,H, Supporting Information), which indicates that the methyltransferase activity is essential for its function on chemoradiotherapy resistance. We then verified the effects of SGC707, a PRMT3‐specific inhibitor,^[^ ^16^ ^]^ on the response of CRC cells to chemoradiation. SGC707 treatment also exhibited an augmented sensitivity to the growth‐suppressive effects of 5‐FU and radiation (Figure S2I–K, Supporting Information). Moreover, SGC707 and 5‐FU exhibit a significant synergistic effect on chemoradiation both in PDOs and CRC cell lines (Figure 2G; Figure S2L, Supporting Information), further suggesting that effects of PRMT3 on chemoradiotherapy were dependent on its enzyme activity.

Conversely, to assess the impact of PRMT3 overexpression (OE) on chemoradiation response, we overexpressed PRMT3 in the HT29 cell line (Figure S2M, Supporting Information), which had the lowest expression of PRMT3 and was most sensitive to radiotherapy and chemotherapy (Figure S1A,B,D, Supporting Information). As expected, we found that *PRMT3*‐OE increased the IC50 of 5‐FU in HT29 cells compared to the CTRL (Figure 2H). Moreover, *PRMT3*‐OE cells demonstrated augmented resilience to the growth‐inhibitory effects of chemoradiation therapy (Figure 2I) and a diminished apoptotic response following 5‐FU and radiation exposure (Figure 2K). In a similar vein, PDOs with *PRMT3*‐OE featured more aggressive growth profiles and heightened resistance, manifesting in larger organoids (Figure 2L–N).

To examine the effect of *PRMT3*‐KO on the chemoradiation resistance in vivo, we subjected tumor‐bearing mice implanted with *PRMT3*‐KO and CTRL SW480 cells to chemoradiation treatment. Our results unambiguously demonstrated that *PRMT3*‐KO tumors were significantly more susceptible to chemoradiation, as attested by reduced tumor sizes and weights (Figure 2O,P; Figure S2N, Supporting Information). Analogous observations were made in PDO‐xenografted mice (Figure 2Q,R; Figure S2O, Supporting Information). Furthermore, *PRMT3*‐OE notably promoted tumor to chemoradiation therapy (Figure 2S,T; Figure S2P, Supporting Information). Taken together, these results indicate that PRMT3 confers chemoradiation resistance of colorectal cancer in vivo and in vitro.

### 2.3 PRMT3 Triggers HR Repair Related Genes and ABC Transporters Expression

**Figure 3:**
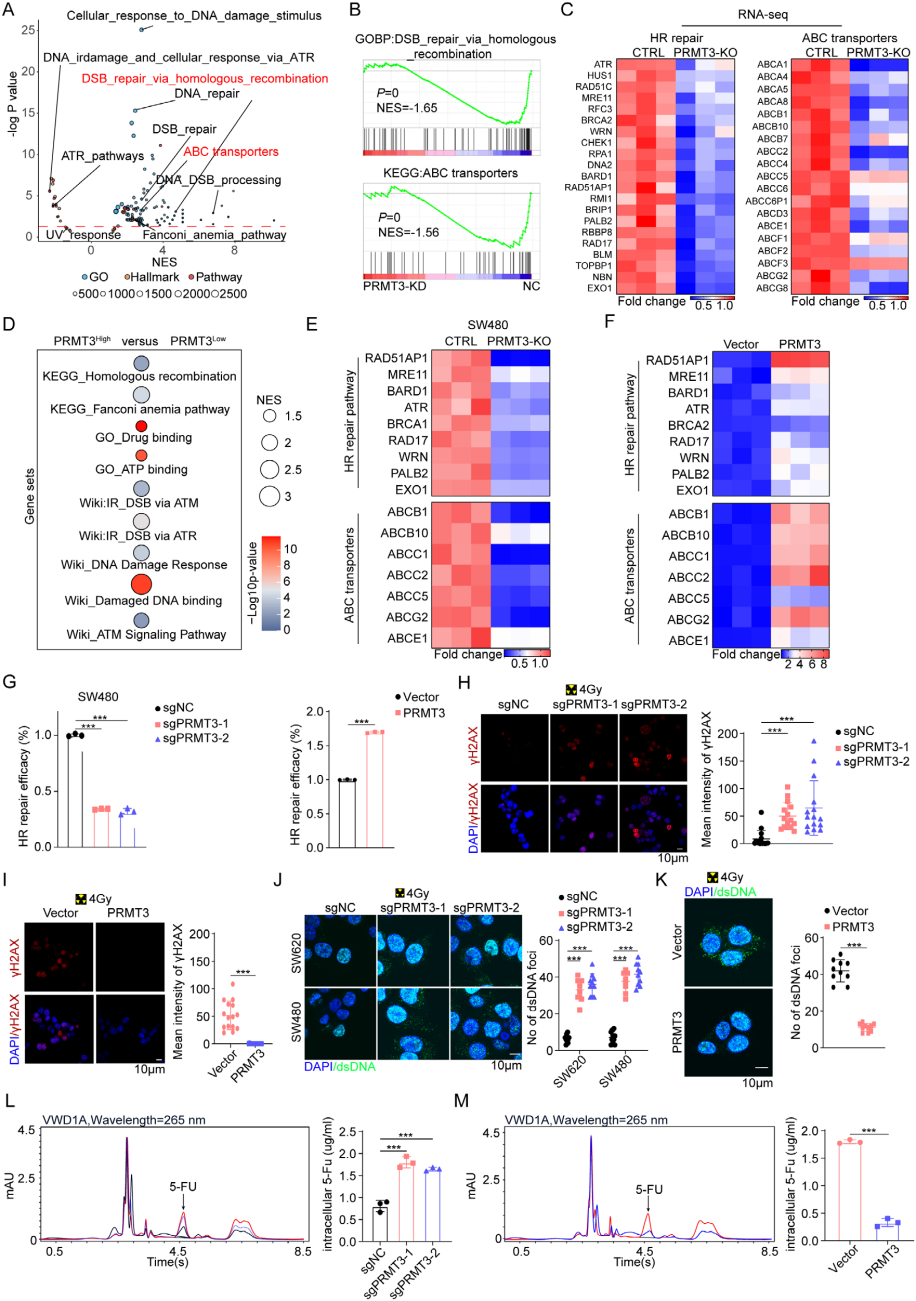
PRMT3 triggers homologous recombination (HR) repair and ABC transporters expression. A) Volcano plots of the pathway enrichment, highlighting the differentially regulated pathways in Ctrl versus *PRMT3*‐KD SW480 cells. FDR, false discovery rate; NES, normalized enrichment score. B) GSEA analysis using the differential expressed genes between Ctrl and *PRMT3*‐KD SW480 cells. C) Heatmap of differential expressed genes between Ctrl and *PRMT3*‐KD SW480 cells which were involved in HR repair and ABC transporters. D) Pathway enrichment using the gene sets containing PRMT3 upregulated and downregulated genes from TCGA‐COAD/READ dataset. E,F) Expression levels of HR‐related genes and ABC transporters in *PRMT3*‐KO versus *PRMT3*‐OE CRC cell lines, measured by qRT‐PCR. G) Quantitative analysis of HR efficiency in *PRMT3*‐KO and *PRMT3*‐OE CRC cell lines. H) Immunofluorescence staining of γH2AX in Ctrl and *PRMT3*‐KO SW480 cells treated with radiation (4Gy). I) Immunofluorescence analysis of γH2AX in Ctrl and *PRMT3*‐OE cells treated with radiation (4Gy). J) Immunofluorescence analysis of dsDNA in Ctrl and *PRMT3*‐KO SW480 cells treated with radiation (4Gy). K) Immunofluorescence analysis of dsDNA in Ctrl and *PRMT3*‐OE cells treated with radiation (4Gy). L) Intracellular 5‐Fu concentration was detected by high‐performance liquid chromatography Ctrl and *PRMT3*‐KO SW480 cells treated with 5‐FU (50 × 10^−6^ m). M) Intracellular 5‐Fu concentration was detected by high‐performance liquid chromatography Ctrl and *PRMT3*‐OE cells treated with 5‐FU (50 × 10^−6^ m).

To elucidate the molecular mechanisms underlying function of PRMT3 in rectal cancer, we performed a transcriptomic analysis of *PRMT3*‐KD SW480 cells and CTRL. The most prevalent altered biological processes were linked to pathways that are involved in DNA repair mechanisms, notably homologous recombination (HR) and ATP‐binding cassette (ABC) transporter activity (**Figure** **3**A–C). Building upon these findings, we conducted a pathway enrichment analysis on transcriptomic datasets derived from The Cancer Genome Atlas’ colorectal adenocarcinoma (TCGA‐COAD) and rectal adenocarcinoma (READ) cohorts. Our analysis consistently demonstrated that samples with elevated PRMT3 expression showed a remarkable emphasis on pathways related to DNA repair and drug resistance (Figure 3D; Figure S3A,B, Supporting Information). We then verified that *PRMT3*‐KO/OE was associated with a downregulation/upregulation of HR repair‐related genes and ABC transporters (Figure 3E,F; Figure S3C,D, Supporting Information).

We then sought to determine whether PRMT3 is involved in the genome damage repair that occurs during radiation. We observed a profound impairment/promotion of HR repair in *PRMT3*‐KO/OE cells (Figure 3G; Figure S3E, Supporting Information). Correspondingly, *PRMT3*‐KO/OE was associated with a significant increased/decreased accumulation of γH2AX and cytoplasmic dsDNA (Figure 3H,I; Figure S3F, Supporting Information), which was indicative of DNA double‐strand breaks. The comet assay supported the same conclusion (Figure S3G, Supporting Information). To probe the impact of PRMT3 on drug efflux mechanisms, we quantified intracellular 5‐FU levels employing high‐performance liquid chromatography (HPLC). In alignment with our preceding observations, *PRMT3*‐KO was accompanied by an elevation in intracellular 5‐FU concentration, whereas *PRMT3*‐OE led to a reduction, implicating PRMT3 in the modulation of drug transport dynamics (Figure 3L,M).

### 2.4 PRMT3 Methylates YBX1 at R247 and Inhibits Its Phase Separation in Cytoplasm

**Figure 4:**
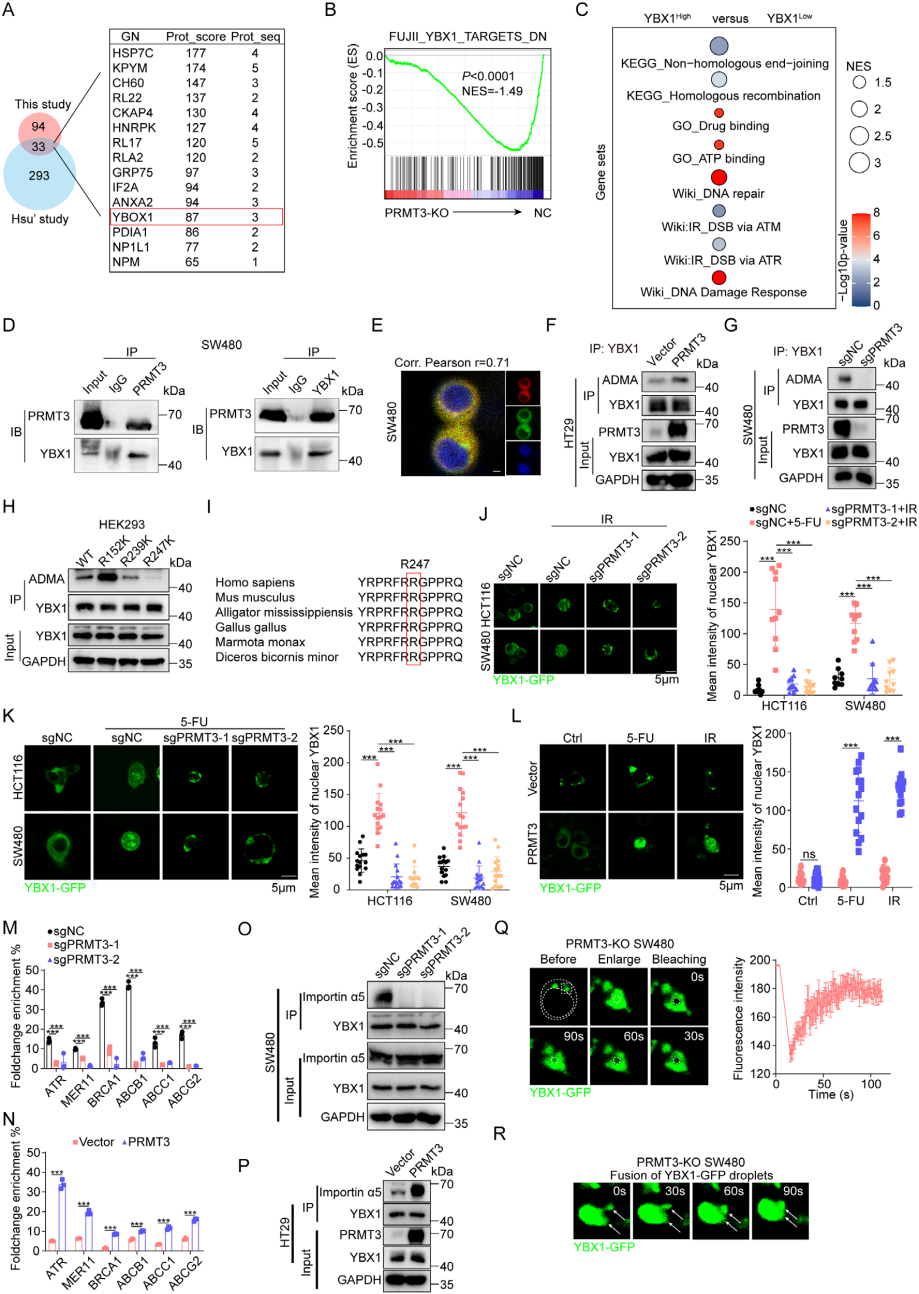
PRMT3 methylates YBX1 at R247 and inhibits its phase separation in cytoplasm. A) Venn diagram showing the 33 common proteins both in our liquid chromatography/tandem mass spectrometry (LC‐MS/MS) and a public dataset. B) GSEA analysis using the differential expressed genes between Ctrl and *PRMT3*‐KD SW480 cells. C) Pathway enrichment using the gene sets containing YBX1 upregulated and downregulated genes from TCGA‐COAD/READ dataset. D) WB analysis showed that endogenous PRMT3 and YBX1 interact with each other in SW480 cells using reciprocal co‐immunoprecipitation. E) Immunofluorescence staining showed the co‐localization of PRMT3 (red) and YBX1 (green) in SW480 cells. Scale bar, 50 mm. F) WB analysis of immunoprecipitated YBX1 to determine the effect of *PRMT3*‐KO on arginine methylation of YBX1 in SW480 cells. G) WB analysis of immunoprecipitated YBX1 to determine the effect of *PRMT3*‐OE on arginine methylation of YBX1 in HT29 cells. H) WB analysis of immunoprecipitated Flag‐tagged YBX1‐WT, YBX1‐R152K, YBX1‐R239K, YBX1‐R247K mutant showed that R247K mutation dramatically reduced ADMA signal in HEK293 cells overexpressing Flag‐tagged YBX1‐WT, YBX1‐R152K, YBX1‐R239K, YBX1‐R247K mutant. I) The sequences surrounding R452 of YBX1 are evolutionarily conserved across multiple species. J,K) The effect of *PRMT3*‐KO on nucleus location of YBX1 in CRC cells with radiation (4 Gy) and 5‐FU (50 × 10^−6^ m) treatment. L) The effect of *PRMT3*‐OE on nucleus location of YBX1 in CRC cells with radiation (4 Gy) and 5‐FU (50 × 10^−6^ m) treatment. M,N) The effect of *PRMT3*‐KO (M)/OE (N) on the DNA binding ability of YBX1 to its targets’ promotors. O) The effect of *PRMT3*‐KO on the interaction between YBX1 and Importin α5. P) Intrinsically disordered region (IDR) prediction of YBX1. (Top) Predictions of prion‐like domains (PrLDs) and disordered regions. (Bottom) Schematic illustration of YBX1 structural domains. Q) (Left) FRAP assays in *PRMT3*‐KO SW480 cells transfected with YBX1‐GFP. (Right) Quantified FRAP rate of each condensate. R) Confocal image sequence showing the fusion and fission between adjacent YBX1‐GFP foci in the cytoplasm of *PRMT3*‐KO SW480 cells.

Considering the functional implications of PRMT3's enzymatic activity as an arginine methyltransferase, we integrated our mass spectrometry dataset from anti‐PRMT3 immuno‐precipitates of HEK293T cell lysates with publicly available mass spectrometry information (Table S2, Supporting Information).^[^ ^17^ ^]^ Notably, among the proteins most frequently co‐precipitated with PRMT3 was YBX1, a known oncogene previously implicated in chemotherapy and radiotherapy resistance through its regulatory roles in HR repair genes and ABC transporters,^[^ ^18^, ^19^ ^]^ was among the top abundantly pulled down proteins by PRMT3 (**Figure** **4**A; Figure S4A, Supporting Information). Importantly, GSEA analysis of *PRMT3*‐KO RNA‐seq data revealed a significant enrichment in YBX1‐Targets pathway (Figure 4B). Complementing this, transcriptomic pathway enrichment in TCGA‐COAD/READ datasets highlighted DNA repair and drug resistance pathways in YBX1‐high tumors (Figure 4C; Figure S4B, Supporting Information). The publicly available CHIP‐seq data revealed the direct binding of YBX1 on promoters of HR repair genes and ABC transporters (Figure S4C, Supporting Information). These findings collectively prompted us to speculate that YBX1 serves as a substrate for PRMT3, mediating its effects on chemoradiation resistance.

To validate our immunoprecipitation‐mass spectrometry (IP‐MS) observations, we assessed the interaction between PRMT3 and YBX1 at the endogenous level. Our results confirmed efficient reciprocal co‐immunoprecipitation of PRMT3 and YBX1 in SW480 and HEK293T cells, supporting a direct physical association (Figure 4D; Figure S4D, Supporting Information). Also, immunofluorescence (IF) staining showed that PRMT3 and YBX1 colocalize in the cytoplasm in SW480 and HEK293T cells (Figure 4E; Figure S4E, Supporting Information). Analysis of YBX1 methylation status upon *PRMT3*‐OE showed an increase in asymmetric dimethylarginine (ADMA) modification of YBX1 in HT29 cells (Figure 4F), while *PRMT3*‐KO/KD led to a significant reduction in YBX1‐ADMA in SW480 and SW620 cells (Figure 4G; Figure S4F, Supporting Information), establishing YBX1 as a PRMT3 substrate.

To pinpoint the specific methylation site, we generated R‐to‐K mutants of YBX1 at the conserved RGG/RG motifs (Figure S4G,H, Supporting Information), given PRMTs’ preference for such sequences.^[^ ^20^ ^]^ Overexpression of YBX1‐wild‐type (WT) or the YBX1‐R247K mutant in HEK293T cells revealed that the R247K mutation abolished YBX1‐ADMA (Figure 4H), signifying R247 as the critical methylation site. Furthermore, the YBX1 amino acid sequence surrounding R247 was evolutionarily conserved across multiple species (Figure 4I), indicating the R247 methylation may be involved in important biological processed.

Given YBX1's nucleus translocation in response to chemoradiation,^[^ ^19^ ^]^ we investigated whether PRMT3 facilitates this process. Indeed, *PRMT3*‐KO hindered YBX1 nucleus localization under 5‐FU and radiation treatments (Figure 4J,K). Conversely, *PRMT3*‐OE in cells with low basal PRMT3 expression promoted YBX1 nucleus shuttling under similar therapeutic conditions (Figure 4L). In tumors with high expression of PRMT3, the proportion of nucleus location of YBX1 was much higher than tumors with low expression of PRMT3 (Figure S4I, Supporting Information). We then demonstrated that *PRMT3*‐KO/OE significantly modulated YBX1 recruitment to the promoters of these genes (Figure 4M,N).

Previous studies have implicated YBX1 phosphorylation in its nucleus transport,^[^ ^21^ ^]^ with phosphorylated YBX1 being escorted to the nucleus by importins. However, we observed no impact of PRMT3 on YBX1 phosphorylation (Figure S4J, Supporting Information). Intriguingly, *PRMT3*‐KO/OE reduced/increased the interaction between YBX1 and importin α5, without affecting importin β binding (Figure 4O,P; Figure S4K, Supporting Information), implicating a physical impediment to YBX1 nucleus translocation. IF analyses suggested that PRMT3 deficiency promoted YBX1 condensation into cytoplasmic droplets (Figure 4J–L), reminiscent of liquid–liquid phase separation (LLPS). Given that arginine methylation, particularly at RGG/RG motifs, can inhibit phase separation,^[^ ^22^ ^]^ and noting YBX1's intrinsically disordered region (IDR) (Figure S4L, Supporting Information), fluorescence recovery after photobleaching (FRAP) assays on YBX1‐containing droplets in SW480 cells revealed rapid recovery dynamics, indicative of LLPS behavior. Fusion events of these droplets were also documented (Figure 4Q,R), confirming their dynamic, liquid‐like properties under PRMT3 depletion. In summary, our findings propose a mechanism by which PRMT3 methylation of YBX1 at R247 inhibits cytoplasmic phase separation, thereby facilitating YBX1's nucleus translocation in response to chemoradiation, with implications for the regulation of DNA repair and drug resistance genes (Figure S4M, Supporting Information).

### 2.5 A Methylation–Demethylation of R247 Relay Spatiotemporally Controls Phase Separation and Transcriptive Activity of YBX1

**Figure 5:**
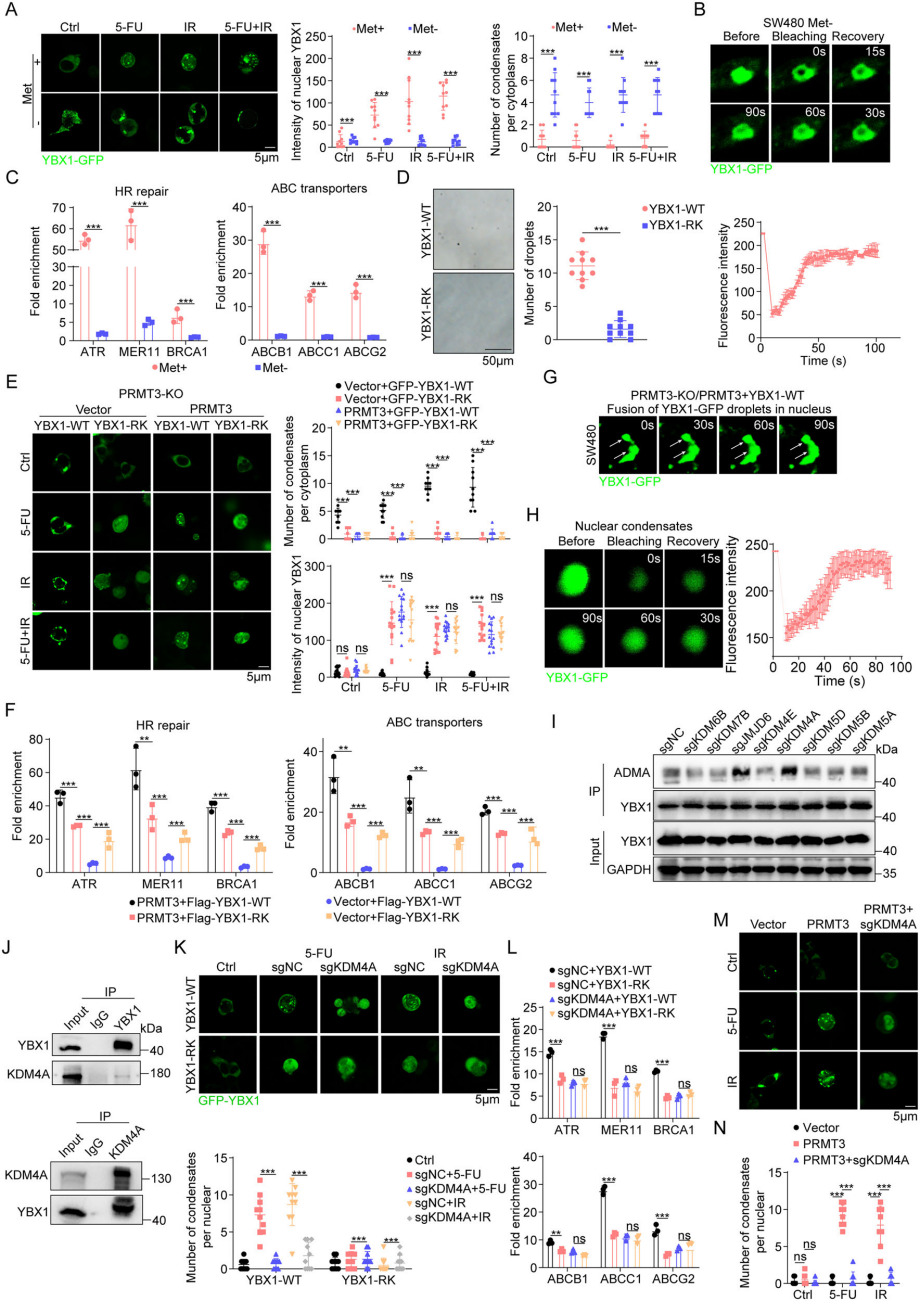
A methylation–demethylation of R247 relay spatiotemporally controls phase separation and transcriptive activity of YBX1. A) (Left) Live imaging of YBX1‐GFP. (Right) Quantification of intensity of nucleus YBX1 and number of condensates in cytoplasm in SW480 cells treated with SAM− or SAM+ culture medium. B) (Upper) FRAP assays in SW480 cells transfected with YBX1‐GFP treated with SAM− culture medium. (Lower) Quantified FRAP rate of each condensate. C) The difference in DNA binding ability of YBX1 to its targets’ promotors between SW480 cells treated with SAM− or SAM+ culture medium was assessed by ChIP assay. D) Representative images of 20 × 10^−6^ m recombinant YBX1‐WT and indicated YBX1‐RK‐GFP protein droplet formation in vitro. E) (Left) Live imaging of YBX1‐GFP and indicated YBX1‐RK‐GFP. (Right) Quantification of intensity of nucleus YBX1 and number of condensates in cytoplasm in indicated *PRMT3*‐KO SW480 cells. F) (Left) FRAP assays in SW480 cells transfected with YBX1‐GFP in indicated *PRMT3*‐KO SW480 cells. (Right) Quantified FRAP rate of each condensate. G) The DNA binding ability of YBX1 in PRMT3‐KO SW480 cells infected with vector + YBX1‐WT, vector + YBX1‐RK mutant, PRMT3 + YBX1‐WT, PRMT3 + YBX1‐RK mutant. H) (Left) FRAP assays in nucleus of SW480 cells transfected with YBX1‐GFP. (Right) Quantified FRAP rate of each nucleus condensate. I) Gene‐editing coupled biochemical screening strategy to discovery candidate arginine methyltransferase(s) responsible for R247 YBX1 demethylation. J) WB analysis showed that endogenous KDM4A and YBX1 interact with each other in SW480 cells using reciprocal co‐immunoprecipitation with radiation (4 Gy) treatment. K) (Upper) Live imaging of YBX1‐GFP and indicated YBX1‐RK‐GFP. (Lower) Number of condensates in nucleus in indicated SW480 cells. L) The DNA binding ability of YBX1 in *KDM4A*‐KO and Ctrl SW480 cells infected with YBX1‐WT and YBX1‐RK mutant was assessed by ChIP assay. M) (Upper) Live imaging of YBX1‐GFP in *PRMT3*‐OE HT29 cells with or without *KDM4A*‐KO. (Lower) Number of condensates in nucleus in indicated HT29 cells. N) The DNA binding ability of YBX1 in *PRMT3*‐OE HT29 cells with or without *KDM4A*‐KO was assessed by ChIP assay.

To elucidate the dependency of YBX1 subcellular localization and phase separation on R247 methylation, we subjected SW480 cells to culture in complete medium versus S‐adenosylmethionine (SAM)‐deficient medium. Under SAM deprivation, YBX1 was retained in the cytoplasm and formed condensates, failing to localize to the nucleus (**Figure** **5**A). FRAP analysis of these YBX1‐containing cytoplasmic droplets revealed rapid recovery kinetics, consistent with LLPS behavior under SAM‐deficient conditions (Figure 5B). Moreover, YBX1 in a SAM‐deprived state did not accumulate at the promoters of its target genes nor effectively upregulate their expression (Figure 5C,D; Figure S5A, Supporting Information). In addition, SGC707 treatment also retained YBX1 in the cytoplasm and formed condensates, failing to localize to the nucleus and modulate targets expression (Figure S5B–E, Supporting Information), which suggested the effects of PRMT3 on YBX1 was dependent on its methyltransferase activity.

To further substantiate the pivotal role of R247 methylation in governing YBX1 localization and phase separation, we purified YBX1‐WT and YBX1‐R247K protein. YBX1‐WT, but not YBX1‐R247K, spontaneously phase separated into droplets upon addition of a crowding reagent (Figure 5D). Given R247 as the exclusive PRMT3‐mediated methylation site, we assessed whether YBX1 function is contingent upon this post‐translational modification. Upon overexpressing YBX1‐R247K or YBX1‐WT in *PRMT3*‐KO SW480 cells and subjecting them to chemoradiation (Figure S5F, Supporting Information), the R247K mutant was more efficiently translocated to the nucleus compared to YBX1‐WT. Intriguingly, co‐expression of PRMT3 with either variant comparably influenced nucleus translocation (Figure 5E). However, YBX1‐WT overexpression in PRMT3‐KO cells promoted cytoplasmic condensate formation, retaining YBX1 within cytoplasmic droplets (Figure 5E). Collectively, these findings imply that only uncovered R247 promoted the LLPS of YBX1 and R247 methylation inhibits the process, thereby facilitating its nucleus import. Notably, we discovered that co‐expression of PRMT3 with YBX1‐R247K resulted in less effective DNA binding and target gene expression compared to co‐expression with YBX1‐WT (Figure 5F; Figure S5G, Supporting Information). To our surprise, the R247F mutant (methylation mimic),^[^ ^23^ ^]^ also ineffectively bind to promoter of target genes and modulate their expression, which indicated the importance of R247, but not only the methylation of YBX1 in the nucleus (Figure S5H–K, Supporting Information).

Importantly, YBX1‐WT, but not YBX1‐R247K/F, once in the nucleus, re‐established condensates, suggesting demethylation‐driven LLPS as indicated by FRAP assay and fusion events (Figure 5G,H; Figure S5L, Supporting Information). Seeking to identify the enzymes responsible for YBX1 demethylation, we generated individual knockout cell lines for previously reported arginine demethylases and found that *JMJD6* and *KDM4A*‐KO enhanced YBX1 methylation (Figure 5I; Figure S6A, Supporting Information). Interaction assays revealed a direct association only between KDM4A and YBX1 (Figure 5J; Figure S6B,C, Supporting Information), implicating KDM4A in YBX1 arginine demethylation. To clarify whether the mechanism by which KDM4A mediates YBX1 demethylation acts directly on the R247 site or through other indirect pathways, we re‐expressed Flag‐YBX1‐WT and Flag‐YBX1‐R247K in the sgNC/sg*KDM4A* SW480 cells. We found that *KDM4A*‐KO notably increased the ADMA level of Flag‐YBX1‐WT compared with the sgNC group. However, *KDM4A*‐KO failed to regulate the ADMA level of Flag‐YBX1‐R247K, which indicated that KDM4A mediates YBX1 demethylation acts directly on the R247 site instead of other indirect pathways (Figure S6D, Supporting Information). YBX1‐WT‐induced nuclear condensates were significantly augmented, and *KDM4A*‐KO suppressed this phenomenon under chemoradiation (Figure 5K). Furthermore, *KDM4A*‐KO negated the heightened promoter occupancy and expression modulation by YBX1‐WT (Figure 5L), implying KDM4A demethylation of YBX1 was essentiality for its condensates re‐establishing and function (Figure S6E,F, Supporting Information). In addition, KDM4A also exhibited closely correlation with ABC transporters or HR repair signature in the data from TCGA‐COAD/READ and our cohort (Figure S6G–J, Supporting Information). It was reported that KDM4A mediated demethylation of H3K9 also modulated gene expression. However, the public ChIP‐seq data exhibited no binding site of H3K9me3 to the promoters of ABC transporters or HR repair genes (Figure S6K, Supporting Information). Furthermore, lower KDM4A expression correlated with enhanced responsiveness to therapy, as confirmed by TRG assessment (Figure S6L, Supporting Information). These data suggested that KDM4A was a positive regulator of ABC transporters or HR repair genes.

Then we explore whether KDM4A mediated the effects of PRMT3. Overexpression of PRMT3 drove YBX1 nucleus translocation and condensate formation, effects partially reversed by *KDM4A*‐KO, as evidenced by a decrease in droplet numbers (Figure 5M). Concomitantly, KDM4A‐KO mitigated the transcriptional activation of YBX1 targets induced by PRMT3‐OE, as reflected in reduced promoter binding and target gene expression (Figure 5N; Figure S6M,N, Supporting Information). In conclusion, our data reveal a spatial and temporal regulation of YBX1 function through a methylation‐demethylation cycle orchestrated by PRMT3 and KDM4A, respectively (Figure S6O, Supporting Information). This intricate interplay dynamically controls YBX1's phase separation and transcriptional activity, underscoring the complexity of epigenetic regulation in cellular responses to stress stimuli like chemoradiation.

### 2.6 A Methylation–Demethylation of YBX1‐R247 Relay is Required for Chemoradiation Resistance

Since that we speculated that methylation–demethylation of YBX1‐R247 relay might contribute to PRMT3‐mediated chemoradiation resistance, we first knocked down YBX1 in SW480 cells (Figure S7A, Supporting Information) and found that *YBX1*‐KD significantly inhibited expression of HR repair genes and ABC transporters, increased γH2AX intensity and inhibited cell growth (Figure S7B–D, Supporting Information). Also, *YBX1*‐KD significantly diminished the effect of PRMT3‐OE on expression of HR repair genes and ABC transporters, γH2AX intensity and cell growth (Figure S7E–G, Supporting Information). However, *YBX1*‐OE in *PRMT3*‐KO SW480 cells did not reverse the effect of *PRMT3*‐KO on expression of HR repair genes and ABC transporters, γH2AX intensity and cell growth (Figure S7H–J, Supporting Information).

**Figure 6:**
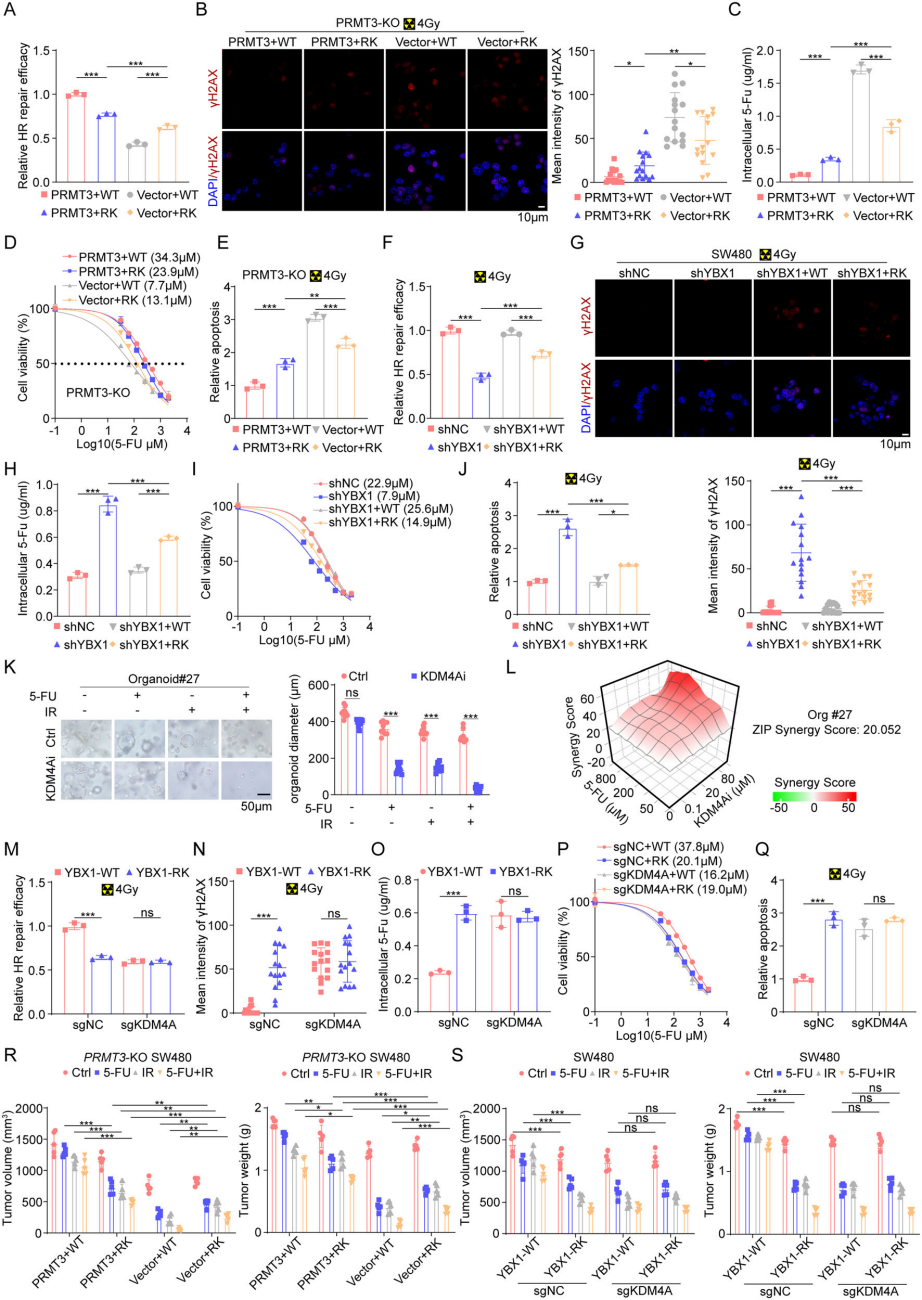
A methylation–demethylation of YBX1‐R247 relay is required for chemoradiation resistance. A) Quantitative analysis of HR efficiency in PRMT3‐KO SW480 cells infected with vector + YBX1‐WT, vector + YBX1‐RK mutant, PRMT3 + YBX1‐WT, PRMT3 + YBX1‐RK mutant. B) Immunofluorescence staining of γH2AX in PRMT3‐KO SW480 cells infected with vector + YBX1‐WT, vector + YBX1‐RK mutant, PRMT3 + YBX1‐WT, PRMT3 + YBX1‐RK mutant. C) Intracellular 5‐Fu concentration in PRMT3‐KO SW480 cells infected with vector + YBX1‐WT, vector + YBX1‐RK mutant, PRMT3 + YBX1‐WT, PRMT3 + YBX1‐RK mutant. D) IC50 of PRMT3‐KO SW480 cells infected with vector + YBX1‐WT, vector + YBX1‐RK mutant, PRMT3 + YBX1‐WT, PRMT3 + YBX1‐RK mutant to 5‐FU. E) Apoptosis of PRMT3‐KO SW480 cells infected with vector + YBX1‐WT, vector + YBX1‐RK mutant, PRMT3 + YBX1‐WT, PRMT3 + YBX1‐RK mutant induced by radiation. F) The effect of YBX1‐WT and YBX1‐RK mutant OE on HR efficiency of YBX1‐KD SW480 cells. G) The effect of YBX1‐WT and YBX1‐RK mutant OE on γH2AX expression of YBX1‐KD SW480 cells. H) The effect of YBX1‐WT and YBX1‐RK mutant OE on intracellular 5‐Fu concentration of YBX1‐KD SW480 cells. I) The effect of YBX1‐WT and YBX1‐RK mutant OE on IC50 of YBX1‐KD SW480 cells to 5‐FU. J) The effect of YBX1‐WT and YBX1‐RK mutant OE on apoptosis of YBX1‐KD SW480 cells induced by radiation (4 Gy). K) The effects of KDM4A inhibitor on growth of PDO#27 after treated with 5‐FU and irradiation (4 Gy). L) Synergy score of PDO#27 treated with KDM4A inhibitor and 5‐FU. M) The effect of YBX1‐WT and YBX1‐RK mutant OE on HR efficiency of *KDM4A*‐KO SW480 cells and control cells. N) The effect of YBX1‐WT and YBX1‐RK mutant OE on γH2AX expression of *KDM4A*‐KO SW480 cells and control cells. O) The effect of YBX1‐WT and YBX1‐RK mutant OE on intracellular 5‐Fu concentration of *KDM4A*‐KO SW480 cells and control cells. P) The effect of YBX1‐WT and YBX1‐RK mutant OE on IC50 of *KDM4A*‐KO and ctrl SW480 cells to 5‐FU. Q) The effect of YBX1‐WT and YBX1‐RK mutant OE on apoptosis of *KDM4A*‐KO and ctrl SW480 cells induced by radiation (4 Gy). R) The measurement of tumor volumes (left) and tumor weight (right) to determine the effect of YBX1‐WT and YBX1‐RK mutant OE on the growth of *PRMT3*‐KO SW480 cells and *PRMT3*‐KO SW480 cells with *PRMT3*‐OE, which were treated with 5‐FU, radiation or combination (*n* = 5). S) The measurement of tumor volumes (left) and tumor weight (right) to determine the effect of YBX1‐WT and YBX1‐RK mutant OE on the growth of *KDM4A*‐KO SW480 cells and ctrl SW480 cells, which were treated with 5‐FU, radiation or combination (*n* = 5).

To scrutinize the regulatory role of R247 methylation in YBX1 function, we assessed the impact of overexpressing the YBX1‐R247K mutant and YBX1‐WT on the responsiveness of *PRMT3*‐KO SW480 cells to combined chemoradiation therapy. Our findings revealed that co‐expression of PRMT3 with the YBX1‐R247K mutant exerted a lesser influence on HR repair efficiency, γH2AX accumulation, and intracellular 5‐FU efflux compared to co‐expression with YBX1‐WT (**Figure** **6**A–C). Conversely, the YBX1‐R247K mutant demonstrated a more pronounced effect than YBX1‐WT in cells devoid of PRMT3 (Figure 6A–C), implicating R247 methylation as crucial for nucleus localization of YBX1 and suggesting that R247 demethylation within the nucleus potentiates its functional role. Moreover, the combination of PRMT3 with the YBX1‐R247K mutant also had a diminished effect on the IC50 of 5‐FU and apoptosis induction under radiation, whereas the YBX1‐R247K mutant surpassed YBX1‐WT in *PRMT3*‐depleted cells (Figure 6D,E). In an effort to deepen our understanding, we contrasted the effects of YBX1‐WT and YBX1‐R247K mutant overexpression on chemoradiation resistance in YBX1‐KD cells. Reintroduction of YBX1‐WT almost entirely restored the HR repair proficiency and intracellular 5‐FU export compromised by *YBX1*‐KD (Figure 6F–H). Conversely, the R247K mutation significantly curtailed YBX1's capability to facilitate HR repair and 5‐FU extrusion (Figure 6F–H). In addition, YBX1‐WT overexpression correlated with a higher IC50 for 5‐FU and reduced apoptosis upon radiation exposure compared to the YBX1‐R247K mutant (Figure 6I,J). These results collectively affirm the indispensability of uncovered arginine 247 for nucleus YBX1's functional integrity.

Proceeding further, we explored whether KDM4A mediates the distinct functions of nucleus YBX1‐WT and the YBX1‐R247K mutant. We found that KDM4A inhibitor significantly inhibited PDOs growth with chemoradiation therapy, increased γH2AX accumulation and apoptosis with radiation, and synergistically acted with 5‐FU (Figure 6K,L; Figure S7K,L, Supporting Information). Importantly, *KDM4A*‐KO virtually abrogated the differential impacts of YBX1‐WT and YBX1‐R247K on HR repair and 5‐FU efflux (Figure 6M–O). Likewise, with KDM4A knocked out, YBX1‐WT and YBX1‐R247K mutant overexpression elicited similar outcomes regarding 5‐FU‐induced cell viability reduction and radiation‐induced apoptosis (Figure 6P,Q; Figure S7K, Supporting Information), thereby validating the necessity of KDM4A‐mediated YBX1‐R247 demethylation for chemoradiation resistance.

Also, we found that *KDM4A*‐KO significantly diminished the effect of *PRMT3*‐OE on cell apoptosis, γH2AX accumulation and tumor growth (Figure S7M–P, Supporting Information). In addition, we observed that co‐expression of PRMT3 with the YBX1‐R247K mutant had a lesser impact on chemoradiation resistance compared to co‐expression with YBX1‐WT (Figure 6R). However, in PRMT3‐depleted tumors, the YBX1‐R247K mutant again outperformed YBX1‐WT (Figure 6R). Analogously, depletion of KDM4A in vivo abolished any discernible difference between YBX1‐WT OE and YBX1‐R247K mutant OE concerning chemoradiation resistance (Figure 6S). Consequently, our findings underscore the vital requirement of a methylation‐demethylation relay at YBX1‐R247 for the development of chemoradiation resistance.

### 2.7 Acipimox Allosterically Inhibited Enzymatic Activity of PRMT3 and Sensitized Colorectal Cancer to Chemoradiation Therapy

**Figure 7:**
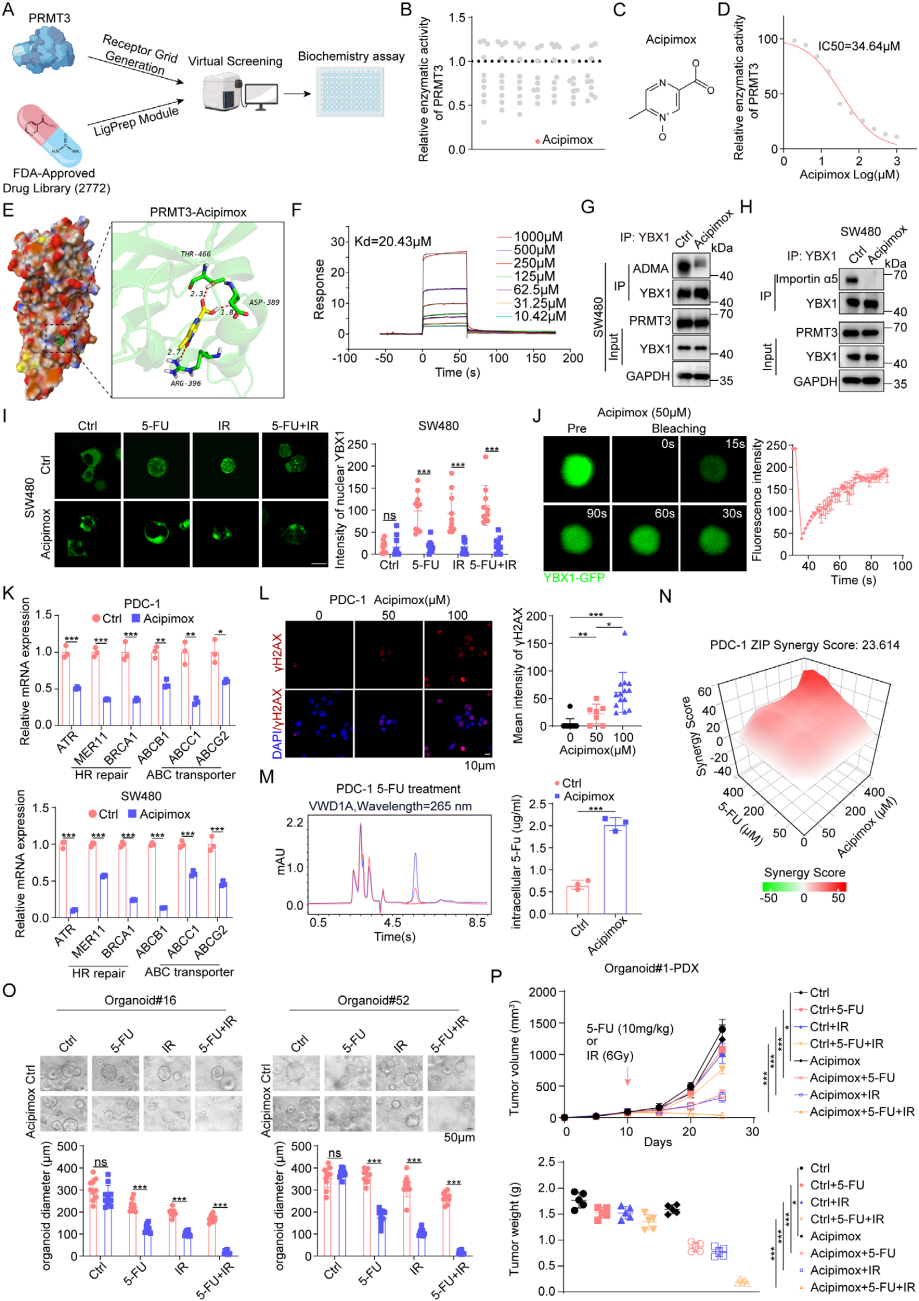
Acipimox allosterically inhibited enzymatic activity of PRMT3 and sensitized colorectal cancer to chemoradiation therapy. A) The flow diagram for PRMT3 inhibitor screening. B) The effect of 69 potential compounds on the enzymatic activity of PRMT3. C) Structure of acipimox. D) IC50 of enzymatic activity of PRMT3 to acipimox. E) Computational model and interactions of acipimox and PRMT3. F) Kinetic constant (KD) analysis of acipimox interacting with PRMT3 using surface plasmon resonance assay. G) The effect of acipimox (50 × 10^−6^ m) on the ADMA level of YBX1 in SW480 cells. H) The effect of acipimox (50 × 10^−6^ m) on the interaction between YBX1 and Importin α5 in SW480 cells. I) The effect of acipimox (50 × 10^−6^ m) on nucleus location of YBX1 in CRC cells with radiation (4 Gy) and 5‐FU (50 × 10^−6^ m) treatment. J) (Left) FRAP assays in acipimox (50 × 10^−6^ m) treated SW480 cells transfected with YBX1‐GFP. (Right) Quantified FRAP rate of each condensate. K) The effect of acipimox (50 × 10^−6^ m) on the expression of HR related genes and ABC transporters in PDC‐1 and SW480 cells. L) Immunofluorescence staining of γH2AX in acipimox (50 × 10^−6^ m) treated PDC‐1 cells under radiation (4 Gy) treatment. M) Intracellular 5‐Fu concentration was detected by high‐performance liquid chromatography in PDC‐1 cells treated with 5‐FU (50 × 10^−6^ m). N) Synergy score of PDC‐1 cells treated with acipimox and 5‐FU. O) The effect of acipimox (50 × 10^−6^ m) on diameter of PDO#16 and #52 after treated with 5‐FU (50 × 10^−6^ m), irradiation (4 Gy) and combination. P) The effects of acipimox (20 mg kg^−1^) on tumor volumes and tumor weights of Organoid#1‐PDX treated with 5‐FU, irradiation and combination.

In pursuit of an alternative to the non‐clinically approved SGC707, we ventured to discover an FDA‐approved compound capable of inhibiting human PRMT3 (PDB ID: 4HSG). Leveraging virtual screening against a library housing 2772 approved drugs, 63 candidates were shortlisted based on docking scores (**Figure** **7**A; Table S3, Supporting Information). Following this, an in vitro enzymatic activity assay narrowed down the selection to potential allosteric inhibitors of PRMT3 (Figure 7A). Ultimately, acipimox emerged as a potent inhibitor of PRMT3's enzymatic activity (Figure 7B–E; Figure S8A, Supporting Information), concurrently reducing global H4R3 methylation levels (Figure S8B, Supporting Information). Surface plasmon resonance assays further confirmed acipimox's high binding affinity to PRMT3, with a Kd value of 20.43 × 10^−6^ m (Figure 7F).

We next investigated whether acipimox could impede the PRMT3‐YBX1 axis. Treatment with acipimox led to a marked reduction in YBX1 arginine methylation (Figure 7G) and disrupted the YBX1‐importin α5 interaction, causing YBX1 to accumulate in cytoplasmic condensates (Figure 7H,I). FRAP analysis attested to these condensates undergoing LLPS under acipimox treatment (Figure 7J). Moreover, acipimox treatment significantly downregulated the expression of HR repair and ABC transporter genes (Figure 7K), thereby efficaciously inhibiting the PRMT3‐YBX1 signaling axis via PRMT3 enzymatic inhibition.

To assess the therapeutic potential of acipimox in conjunction with chemoradiation for colorectal cancer, we employed human organoids, patient‐derived cells (PDCs), and CRC cell lines. The combination of acipimox and radiation significantly augmented DNA damage, as evidenced by increased nucleus γH2AX and cytoplasmic dsDNA levels in a dose‐dependent fashion (Figure 7L; Figure S8C,D, Supporting Information). Acipimox also inhibited 5‐FU efflux in PDCs and synergistically acted with 5‐FU (Figure 7M,N). This combination regimen displayed superior therapeutic efficacy across PDCs, CRC cell lines, and PDO models (Figure 7O; Figure S8E–H, Supporting Information). Additionally, in patient‐derived xenograft (PDX) model harboring high PRMT3 expression, while acipimox or chemoradiation alone had limited impact, their combination elicited substantial tumor regression (Figure 7P; Figure S8I, Supporting Information). Notably, the combination treatment with acipimox did not lead to a significant reduction in mouse weight, which preliminarily suggests a favorable safety profile of acipimox (Figure S8J, Supporting Information). These data cumulatively illustrate that the acipimox‐chemoradiation combination markedly enhances therapeutic effectiveness, posing as a promising strategy for CRC treatment.

Given the reported enhancement of immunotherapy efficacy by neoadjuvant chemoradiation in rectal cancer, yet with only approximately 20% of patients benefiting due to resistance mechanisms,^[^ ^4^ ^]^ we probed whether acipimox could augment the immunotherapeutic potential of chemoradiation. Intriguingly, while chemoradiation alone failed to sensitize the CT26 tumor‐bearing model to anti‐PD1 immunotherapy, acipimox dramatically enhanced the sensitivity of these tumors to the combination of chemoradiation and anti‐PD1 therapy (Figure S7K–M, Supporting Information). Enhanced infiltration of CD8^+^ T cells and cytotoxic immune cells were observed in acipimox treated CT26 tumors from immunocompetent hosts (Figure S7N, Supporting Information). Collectively, our study proposes acipimox as a potent sensitizer for chemoradiation‐anti‐PD1 immunotherapy combinations, broadening the therapeutic horizon for CRC management.

## 3 Discussion

Despite the proliferation of diverse therapeutic strategies, such as immunotherapy, in the management of locally advanced rectal cancer, neoadjuvant chemoradiotherapy persists as the cornerstone of treatment.^[^ ^3^ ^]^ However, the suboptimal response rates culminate in unfavorable prognoses for a subset of patients.^[^ ^4^ ^]^ This work pioneers the exploration of arginine methylation's role in chemoradiotherapy resistance and introduces, for the first time, an FDA‐approved drug capable of potentiating the efficacy of neoadjuvant chemoradiotherapy. Our findings delineate a spatiotemporal methylation‐demethylation relay of YBX1, orchestrated by PRMT3 and KDM4A, which governs phase separation dynamics and chemoradiotherapy responsiveness, exemplifying the intricate coordination of biological regulation in malignancy (Figure S8, Supporting Information).

Considering the low pathologic complete response (pCR) rate associated with neoadjuvant chemoradiotherapy in this patient population, the quest for an efficacious biomarker to identify those likely to benefit from conventional treatment is imperative. Through functional screenings, we have identified PRMT3 as a pivotal mediator of chemoradiotherapy resistance and a strong correlate of therapeutic outcome. This correlation was corroborated in rectal cancer patient samples and PDO models. Our data suggest that elevated PRMT3 expression inversely associates with treatment response, overall survival, and progression‐free survival in neoadjuvant settings, positioning PRMT3 as a promising biomarker candidate for tailored neoadjuvant therapies in rectal cancer. Clinical implementation could involve utilizing pretreatment biopsy PRMT3 expression levels to inform personalized treatment strategies. Nonetheless, further validation with a larger patient cohort is warranted to ascertain PRMT3's predictive utility in neoadjuvant therapy response.

Previous research from our group implicated PRMT3 in oxaliplatin resistance through IGF2BP1 methylation,^[^ ^13^ ^]^ with IGF2BP1 being implicated in chemoresistance across several cancer types.^[^ ^24^, ^25^, ^26^ ^]^ We speculate IGF2BP1 may partly mediates chemoresistance in rectal cancer. Further analysis indicated that PRMT3 was obviously involved in HR repair and ABC transporters, which was closely related to chemoradiotherapy.^[^ ^27^, ^28^, ^29^ ^]^ However, IGF2BP1 had not been reported to regulate expressions of HR repair‐related genes and ABC transporters ever. Conversely, YBX1 is recognized for its transcriptional control over HR repair genes and ABC transporters in cancer,^[^ ^18^, ^19^ ^]^ leading us to posit that YBX1, rather than IGF2BP1, is the critical substrate through which PRMT3 regulates chemoradiotherapy resistance in rectal cancer. Future endeavors must elucidate the potential contributions of additional PRMT3 targets to this resistance mechanism.

Numerous methyltransferases, encompassing those from the PRMT, KMT, KAT, and HDAC families, have been associated with drug resistance.^[^ ^30^ ^]^ Methyltransferases typically modulate substrate methylation to alter their function. The majority of them govern treatment resistance by modifying histone methylation and consequently influencing gene expression.^[^ ^31^ ^]^ However, PRMT3 primarily resides in the cytoplasm and mainly methylates cytoplasmic proteins. It rarely affects drug resistance through histone modifications. For instance, PRMT3 promotes liver cancer growth by methylating LDHA, confers oxaliplatin resistance by methylating IGF2BP1, and mediates immunotherapy resistance by methylating HSP60.^[^ ^13^, ^32^, ^33^ ^]^ In this study, PRMT3's methylation of YBX1 also occurred in the cytoplasm. This unique distribution may allow PRMT3 to influence protein translation, metabolism, and cellular distribution, thereby impacting drug resistance. Future research should explore more PRMT3 substrates and functions in the cytoplasm.

YBX1, a prominent oncogene implicated in therapy resistance across multiple cancer modalities (e.g., chemotherapy, radiotherapy, targeted therapy and immunotherapy),^[^ ^34^, ^35^, ^36^, ^37^ ^]^ undergoes various post‐translational modifications (PTMs), including phosphorylation, ubiquitylation, acetylation, and methylation, each playing a pivotal role in its diverse biological functions.^[^ ^38^ ^]^ S102 phosphorylation, for instance, facilitates YBX1's nucleus translocation and DNA binding.^[^ ^39^ ^]^ Other YBX1 phosphorylation sites have also been identified to regulate its function and localization.^[^ ^40^, ^41^ ^]^ Ubiquitylation controls various cellular processes through proteasomal degradation of YBX1.^[^ ^42^ ^]^ Residues R199, 200, and 239 of YBX1 were identified as methylated sites using immunoaffinity purification of peptides followed by MS analysis.^[^ ^43^ ^]^ Previous study uncovered that R205 of YBX1 was methylated by PRMT5 and promoted its interaction with p65 in colorectal cancer.^[^ ^44^ ^]^ Here, we identified a novel asymmetric demethylation site of YBX1, R247, which methylated by PRMT3, and was required for its nucleus import by inhibiting its phase separation in cytoplasm. Also, the YBX1 amino acid sequence surrounding R247 was evolutionarily conserved across multiple species. Previous study found that the RG/RGG regions contributed to the formation of protein condensates and arginine methylation at the RG/RGG‐rich regions significantly impair LLPS capability.^[^ ^22^, ^45^, ^46^ ^]^ Our data indicated that R247 was required for phase separation of YBX1 in cytoplasm. Furthermore, the YBX1 condensate significantly isolates its interaction with importin a5, which restricts its nucleus localization under chemoradiation treatment. The asymmetric demethylation counteracts this effect, disrupts the phase separation in the cytoplasm and promotes its nucleus transport as a defense mechanism. Interestingly, once YBX1 was transported into nucleus, the R247 residue would be unmasked and the condensate reformed, which strengthened its ability to transcriptionally regulate expressions of ABC transporters. The interplay between methylation status and YBX1's subcellular localization, as well as its transcriptional regulatory function, underscores the sophisticated regulatory machinery underlying cellular processes, akin to the palmitoylation‐depalmitoylation relay controlling GSDMD activation in pyroptosis.^[^ ^47^ ^]^ Such a stands as a testament to the profound complexity and refined elegance that underpin the myriad phenomena governing life at its fundamental level. YBX1 phase separation plays crucial physiological roles. It drives the formation of membraneless organelles like stress granules and P‐bodies, enabling YBX1 to dynamically assemble with other RNA‐binding proteins and RNAs.^[^ ^48^ ^]^ This process facilitates the regulation of mRNA metabolism, including storage, stabilization, and translation. In addition, it contributes to transcription‐related complex formation, influencing gene expression.^[^ ^49^ ^]^ However, this study reveals that hypomethylation of YBX1's arginine residues promotes its phase separation in the cytoplasm, inhibits binding to nuclear transport proteins, and suppresses nuclear import and transcriptional functions. This uncovers a novel function of YBX1 phase separation. Future research will continue to focus on the role of phase separation in cancer treatment resistance.

As a lysine methyltransferase, JMJD6 demethylates both histone and non‐histone proteins.^[^ ^50^ ^]^ Thus, we hypothesize that acipimox might exert an indirect influence on the arginine methylation of YBX1 through two distinct mechanisms. First, JMJD6 may demethylated proteins related to YBX1, which further affecting the arginine methylation of YBX1. Second, JMJD6 might modulate gene expression including other methyltransferases by demethylates histones and further influence signaling pathways or other molecular pathways, which was related to the arginine methylation of YBX1.

Acipimox, a clinically established hypolipidemic agent for non‐responsive patients,^[^ ^51^ ^]^ exerts its action through lipolysis inhibition and free fatty acid flux reduction, impacting hepatic very‐low‐density lipoprotein (VLDL) synthesis and plasma lipid profiles.^[^ ^52^ ^]^ Here, we found that acipimox improved therapeutic efficacy by inhibiting PRMT3 in rectal cancer. Given the intimate association between fatty acid metabolism and tumors,^[^ ^53^, ^54^, ^55^ ^]^ acipimox potentially exerts an influence on the efficacy of chemoradiotherapy via fatty acid metabolism‐related pathways. As an antilipolytic drug, the role of acipimox in tumors is not fully understood. Previous study indicated that it curbs postprandial glycogen synthesis and gluconeogenesis, and reduces plasma fatty acid levels.^[^ ^56^ ^]^ We speculate this might indirectly suppress tumor metabolism and growth, and influence treatment resistance. In addition, study showed acipimox increased tumor uptake of (18)F‐FDG,^[^ ^57^ ^]^ suggesting a potential impact on tumor metabolism and growth. As an FDA‐approved medication, acipimox presents a translational opportunity to augment chemoradiotherapy responsiveness.

This study has several limitations. First, in clinical settings, patients with rectal cancer typically undergo multi‐drug chemotherapy regimens anchored by 5‐FU. However, our study solely employed 5‐FU as a representative agent, which may not fully mirror the real‐world clinical treatment landscape. Moving forward, we will continue our efforts to develop a more refined neoadjuvant therapy model for colorectal cancer. Second, the safety assessment of acipimox in combination with chemoradiotherapy was based merely on monitoring changes in mouse body weight. Future studies should incorporate a broader range of parameters, including hepatic and renal function, behavioral status, and neurological performance, to achieve a more comprehensive evaluation of safety. Third, while our study confirmed through in vivo and in vitro experiments that acipimox binds to PRMT3 and modulates its activity, it is unclear whether acipimox can interact with other methyltransferases. Our ongoing research will delve deeper into the interactions between acipimox and other methyltransferases to elucidate these relationships more fully.

## 4 Conclusion

In summary, we identify PRMT3 as a key driver of chemoradiotherapy resistance in rectal cancer. PRMT3 induces the arginine methylation of YBX1, which suppresses YBX1's capacity for phase separation in cytoplasm and is imperative for its nucleus translocation. KDM4A demethylates YBX1 to unmask the R247 residue and promote its phase separation in the nucleus, which strengthens its ability to transcriptionally regulate expressions of ABC transporters and HR repair genes and reinforces chemoradiotherapy resistance. High‐throughput FDA approved drugs library screening identifies acipimox as a potent PRMT3 inhibitor and chemoradiotherapy sensitizer. This study offers a tangible prospect for improving therapeutic outcomes in rectal cancer patients in a clinically relevant setting.

## 5 Experimental Section

### Patients and Tissues

The usage of rectal samples was approved by the Ethics Committee of the Sixth Affiliated Hospital, Sun Yat‐Sen University (Ethics Approval ID: L2024ZSLYEC‐117). Written informed consent was obtained from each patient. The study design conformed to the ethical guidelines of the 1975 Declaration of Helsinki. Human rectal tissues were obtained from patients who received curative surgery at the Sixth Affiliated Hospital, Sun Yat‐Sen University. Sex was not considered as a biological variable. The neoadjuvant chemoradiotherapy cohort and neoadjuvant chemotherapy cohort were used for survival analysis and treatment response evaluation, were collected from January 2009 to May 2020. Based on the median IHC scores, we then divided these patients into PRMT3‐High group and PRMT3‐Low group.

### Evaluation of Treatment Efficacy

OS was defined as the interval from date of surgery to date of death or last follow‐up. The TRG score system was used to evaluate the response to neoadjuvant therapy. This grade describes the proportion of primary tumor mass in the resection specimen that is replaced by fibrosis after neoadjuvant treatment. It is graded on a five‐point scale from TRG1 (complete response: 100% fibrosis, no viable tumor cells) to TRG5 (absence of response: no fibrosis, 100% viable tumor cells).^[^ ^58^ ^]^

### Functional Screening

Primary screening was performed using a lentiviral single‐guide RNA (sgRNA) library. A detailed gene list and target sequences are provided in Table S1 (Supporting Information). Cells were infected with lentivirus in six‐well plates. Forty‐eight hours after infection, cells were selected with puromycin (2 µg mL^−1^) for 3 d. Then cells were seeded into 96‐well plates in five replicates and treated with vehicle, 5‐FU (6 × 10^−3^ m), or radiation (8 Gy) for 5 d. The cell viability was evaluated with CCK‐8 assay.

### Lentivirus Virus Production and Infection

The sgRNAs and shRNAs for gene candidates were designed using the MIT online tool CRISPRPICK (Tables S1 and S4, Supporting Information). HEK293T cells were seeded in 6 cm plate and transfected with 2.5 mg lentiCRISPRv2 KO or lentiCRISPRv2 control plasmids, 5 mg psPAX2, and 2.5 mg pVSV‐G plasmids using Lipofectamine 2000 to produce lentivirus. The supernatants containing lentivirus were harvested and filtered 72 h post‐transfection.

### Tissue Dissociation

For tissue dissociation, the fresh tissues were first cut into 1–3 mm^3^ pieces. Then the tissues were digested in digestion medium (Absin, abs9445) on a shaker at 37 °C for 30 min with occasional pipetting until the visible pieces disappeared. Dissociated cell clusters were centrifuged at 400 *g* for 4 min, washed once with organoid culture buffer (Absin, abs9445), and spun down again at 400 *g* for 4 min. If the pellet showed a visible red color, erythrolysis was performed with RBC lysis buffer (eBiosciences, #00‐4333‐57) before the washing step.

### Organoid Culture and Passage

Dissociated cell clusters were resuspended in cold Matrigel (Corning, #356237) and seeded in a prewarmed 24‐well plate at 25 µL drops. The drops were solidified in a 37 °C and 5% CO_2_ incubator for 30 min, and then 1 mL organoid culture medium (Absin, abs9445) was added to each well and refreshed every 2–3 d. Passaging was performed every 1–3 weeks based on organoid density and size. After removing the culture medium, organoids were resuspended in cold trypsin (Absin, abs9445) by pipetting, and then incubated at 37 °C for 4 min. Additional pipetting and incubation were performed if needed. Subsequently, the cells were centrifuged at 400 *g* for 4 min, washed with 12 mL organoid culture buffer, and centrifuge again. The cells were resuspended in Matrigel at a ratio of 1:3, seeded in the plates and cultured as described above.

### Cell Proliferation, Colony Formation, and Apoptosis Assays

For the cell proliferation assay, 1000 cells were seeded into 96‐well plates, cell viability was assessed for five consecutive days with or without 5‐FU/IR treatment by the Cell Counting Kit‐8 (CCK‐8) (Dojindo, Japan). For the colony formation assay, 1000 cells were seeded into six‐well plates for about 10 d with or without 5‐FU/IR treatment, which were stained with crystal violet and counted at the endpoint. All studies were conducted in triplicates. For the apoptosis assay, cells were treated with 5‐FU/IR for 48 h. The cells were labeled with Annexin V/APC and 7‐AAD (KeyGEN BioTECH, China) according to the manufacturer's instructions, the gating strategy for apoptosis assay measured by flow cytometry was shown in Figure S9 (Supporting Information).

### Immunoblotting (IB)

Cells and tissues were lysed with RIPA lysis buffer. Proteins were extracted and loaded in SDS‐PAGE, and transferred onto PVDF membrane (Millipore, Billerica, MA). After blocking with 5% skim milk (Beyotime, China) and sequential incubation with the primary and secondary antibodies (Table S5, Supporting Information), the blots were detected using the ECL detection kit (Millipore).

### Quantitative RT Real‐Time PCR (qPCR)

Total RNA extraction and cDNA synthesis were performed using RNA‐Quick Purification Kit (ES Science, Guangzhou, China) and Prime‐Script cDNA synthesis kits (Invitrogen, California, USA) according to the manufacturer's instructions. The cDNA products were used for qPCR analysis using SYBR Green PCR kit (Invitrogen, California, USA). Table S6 (Supporting Information) includes detailed information about the sequence of the used primers.

### HPLC Analysis of Intracellular 5‐FU

5‐FU ranged from 0.2 to 50 mg L^−1^ for preparation of the standard curves. Cells were inoculated in the six‐well plate then treated with 5‐FU (25 mg L^−1^) for 6 h. After cells counting, CRC cells were resuspended in 150 µL PBS and lysed by sonication in ice‐water bath for 30 min. The procedure of solid‐phase extraction was utilized with styrene‐divinylbenzene resin SPE columns as shown before. Then the samples were centrifuged at 10 000 *g* for 15 min. The fresh supernatants were filtered and injected into HPLC (Waters QDA) for analysis. The concentrations of 5‐FU were calculated by the respective calibration curves.

### Immunohistochemical (IHC) of Clinical Rectal Cancer Specimens

Paraffin‐embedded HCC tissues were cut into 5‐mm sections. The sections were subsequently incubated with antibodies against human PRMT3 (Abcam, Ab191562, 1:200). The IHC score was determined by immunohistochemical staining intensity and the percentage of positively stained cells. Firstly, staining intensity, reflecting the color depth of the immunoreaction, was assessed subjectively under a microscope and graded on a scale of 0 to 3: 0 for no staining, 1 for weak staining, 2 for moderate staining, and 3 for strong staining. Subsequently, the percentage of positively stained cells, indicative of the extent of protein expression within the tissue sample, was estimated and recorded as a value ranging from 0 to 100% (0–25%, 1; 26%–50%, 2; 51%–75%, 3; 76%–100%, 4). The IHC score was then calculated by multiplying the staining intensity score by the percentage of positive cells. Immunohistochemical results were evaluated by two independent observers who were blinded to the clinical outcomes.

### Immunoprecipitation (IP), Mass spectrometry Analysis of PRMT3 Interacting Proteins, and YBX1 Arginine Methylation

Cells and tissues were lysed using IP lysis buffer (Thermo Fisher Scientific, #87787) supplemented with proteinase inhibitor (Sigma‐Aldrich, #04693132001) for 10 min, and cleared by centrifugation at 12 000× *g* at 4 °C for 20 min. Cell lysate (2 mg) was subjected to IP with the indicated antibodies overnight at 4 °C. Then the lysate was incubated with protein A/G agarose beads (Thermo Fisher Scientific, #88803) for 1 h at room temperature. The beads were washed three times with IP lyse buffer. Then the proteins were eluted with loading buffer and detected by western blotting. For mass spectrum analysis of PRMT3 interaction, the peptides were extracted and evaporated for liquid chromatography‐mass spectrometry (LC‐MS) analysis at the FITGENE company (Guangzhou, China). After sample digestion and ionization, generated spectra were matched to a protein database. The scoring algorithms evaluate the match between experimental and theoretical spectra, assigning a score based on factors like peptide matches, fragmentation pattern similarity, and protein coverage. A higher score indicates a more confident protein identification.

### Immunofluorescent

For IF analysis of cultured cells, cells were grown on chamber slides precoated with poly(l‐lysine). Cells were fixed with cold paraformaldehyde. Cultured cells were permeabilized with PBS containing 0.1% Triton X‐100, and blocked with AquaBlock (East Coast Bio, North Berwick, ME). Cells were probed with the following primary antibodies as following: anti‐PRMT3 (Proteintech, 17628‐1‐AP, 1:200), anti‐YBX1 (Proteintech, 20339‐1‐AP, 1:200), γH2AX (Proteintech, 83307‐2‐RR, 1:200), and dsDNA (Sigma‐Aldrich, ZRB2042, 1:200) (Table S5, Supporting Information). After washing the cells with PBS‐T three times, the cells were incubated with 594 (or 488) labeled secondary antibodies (1:200) and DAPI‐containing mounting solution VECTASHIELD (Vector Laboratories). The slices were visualized by using a Nikon inverted microscope Eclipse Ti‐U equipped with a digital camera, or a Nikon A1 laser scanning confocal microscope at the Center for Advanced Microscopy/Nikon Imaging Center (CAM).

### In Vitro Phase Separation Assay

In vitro phase separation assay was performed in storage buffer with indicated protein concentrations, and PEG8000 was also added to a final concentration of 10% (w/v). Phase separation assay was carried out on glass‐bottomed dishes, sealed with optically clear adhesive film to prevent evaporation and observed under an Olympus FV3000 confocal microscope equipped with 60× oil immersion objectives. The phase separation assay using YBX1‐GFP was performed in a physiological LLPS buffer (20 × 10^−3^ m Tris‐HCl, pH 7.5, 15 × 10^−3^ m NaCl, 130 × 10^−3^ m KCl, 5 × 10^−3^ m KH_2_PO_4_, 1.5 × 10^−3^ m MgCl_2_, and 1 mg mL^−1^ BSA).

### Fluorescence Recovery after Photobleaching

The FRAP assays were conducted using the bleaching module of the Zeiss LSM 880 confocal microscope for GFP‐YBX1 droplets individually. The 488 nm laser was used to bleach the EGFP signal. Bleaching was focused on a circular region of interest (ROI) using 100% laser power, and time‐lapse images were collected afterward. A same‐sized circular area away from the bleaching point was selected as an unbleached control. The fluorescence intensity was directly measured in the Zen software. The values were reported as relative to pre‐bleaching time points. GraphPad Prism (version 8.0) was used to plot the data.

### The Homologous Recombination Efficiency Assessment

Cells were cultured in appropriate cell culture media until about 70%–80% confluence. Then the cells were transfected with the HR reporter plasmid (DR‐GFP) and the I‐SceI expression plasmid. The I‐SceI endonuclease will introduce a DSB in the DR‐GFP reporter plasmid, and the efficiency of HR repair can be assessed by flow cytometer to quantify the proportion of GFP+ cells.

### Chromatin‐Immunoprecipitation‐Quantitative PCR (ChIP‐qPCR)

ChIP assays were performed with a ChIP kit (Cell Signaling Technology, Boston, USA, 2952825) according to the manufacturer's instruments. Briefly, 1% formaldehyde (Sigma‐Aldrich, Germany) solution was added to induce SW480 and HCT116 cells crosslinking followed by glycine solution to quench the reaction. Afterward, the cells were lysed, and the nucleoprotein complexes were sonicated for 10 cycles of 10 s power‐on and 20 s intervals with an intensity of 200 W with the sonicate conductor (Qsonica, USA). Then, anti‐STAT1 antibody or IgG was added and incubated with the complexes overnight at 4 °C. The next day, Protein A/G magnetic beads were added to precipitate the indicated fragments for an additional 4 h at 4 °C. After extraction and purification of the indicated DNA, semiquantitative PCR was performed to identify the region interacting with the targets‐specific primers. The experiments were performed in triplicate and the amount of immunoprecipitated DNA was normalized to the input.

### Virtual Screening of Small Molecule Compounds Targeting Human PRMT3

*Protein Preparation*: Retrieve the three‐dimensional structure of Human PRMT3 (PDB ID: 4HSG) from the RCSB PDB database. Subsequently, employ the Protein Preparation Wizard module to refine the protein structure. This refinement process involves the addition of hydrogen atoms, the removal of water molecules, and the optimization of the energy state using the OPLS2005 force field with a convergence criterion of 0.3 Å RMSD. Furthermore, utilize the Receptor Grid Generation module to generate a grid file centered on the small molecule ligand, with a cubic box size of 20 Å.

*Compound Preparation*: Subject the 2D structures of the FDA‐Approved Drug Library, comprising 2772 compounds, to preprocessing using the LigPrep Module of the Schrödinger software. This process includes the addition of hydrogen atoms, energy optimization, and other preparatory steps to obtain suitable 3D structures for subsequent virtual screening.

*Molecular Docking*: Execute virtual screening utilizing the Virtual Screening Workflow module. Import the preprocessed compounds and engage the Glide module for molecular docking. This process entails geometrically and energetically aligning the receptor and ligand molecules. Initially, apply the Standard Precision (SP) mode to filter the 2772 small molecule compounds from the FDA‐Approved Drug Library. Based on scoring, select the top 20% of compounds and further scrutinize them through a third round of screening employing the Extra Precision (XP) mode to establish a ranked list of small molecule candidates. Subsequently, conduct a manual evaluation of the binding affinity between the target and the compounds, along with an assessment of their structural features, to identify the top 200 compounds from the FDA‐Approved Drug Library for further analysis and output.

### PRMT3 Enzymatic Activity Evaluation

PRMT3 enzymatic activity was detected with the SAM510: SAM Methyltransferase Assay (G‐Biosciences, #786‐430). Briefly, a total volume of 5 µL of SAM methyltransferase samples was aliquoted to at least two wells of a 96‐well plate. Then 10 µL the appropriate acceptor substrate was added to the sample and background control. In addition, immediately prior to use and in a suitable tube, prepare the SAM Methyltransferase Assay Master Mix according to the instruction. Then initiate the reaction by adding 100 µL SAM Methyltransferase Master Mix to the wells. Immediately, zero the wells and begin measuring the absorbances at 510 nm collecting data every 10–30 s at 37°C until the increasing absorbances plateau (≈15–30 min).

### Surface Plasmon Resonance (SPR)

During the experimental procedure, 1×HBS‐EP+ buffer (comprising 1 × 10^−3^ m HEPES, 150 × 10^−3^ m NaCl, 3 × 10^−3^ m EDTA, 0.05% surfactant P20, pH 7.4) was utilized as the running buffer. Two channels on the CM5 chip were selected for the experiment. A freshly mixed solution of 50 × 10^−3^ m N‐hydroxysuccinimide (NHS) and 200 × 10^−3^ m 1‐ethyl‐3‐(3‐dimethylaminopropyl) carbodiimide hydrochloride (EDC) in a 1:1 ratio was injected into channel 2 at a flow rate of 10 µL min^−1^ for 900 s. The target protein was diluted to 100 µg mL^−1^ using 10 × 10^−3^ m Acetate 4.5 and immobilized onto the chip surface, achieving a coupling level of ≈22 900 RU. Subsequently, 1 m ethanolamine was injected at a flow rate of 10 µL min^−1^ for 900 s to deactivate any remaining reactive sites. The protein‐coupled chip was then equilibrated at a flow rate of 10 µL min^−1^.

Using the same 1×HBS‐EP+ buffer, the binding and dissociation of acipimox compounds and Recombinant PRMT3 protein were investigated at varying concentrations in sequential injection cycles. Channel 1 served as a blank reference channel. The diluted acipimox compounds were injected consecutively into channels 1 and 2 at concentrations ranging from 10.42 to 1000 × 10^−6^ m, with a flow rate of 30 µL min^−1^. Each injection cycle comprised a binding phase of 60 s and a dissociation phase of 120 s. The experiments were conducted at a controlled temperature of 25 °C.

The affinity between acipimox compounds and Recombinant PRMT3 protein was analyzed using the T200 analysis software (version 3.2) in a 1:1 Binding analysis mode. The affinity constant (KD) was determined to quantify the binding affinity.

### Animal Experiments

All animal experiments were approved by the Institutional Animal Care and Use Committee of the Sixth Affiliated Hospital, Sun Yat‐Sen University (Ethics Approval ID: L2024ZSLYEC‐117). BALB/C nude mice were kept in an animal room with a 12‐h light‐dark cycle at a temperature of 20–22 °C with 40%–70% humidity. For the subcutaneous tumor models, 5 × 10^6^ SW480 or HCT116 cells were injected into four‐week‐old male BALB/C nude mice, and the tumor tissues were taken out one month later for future experiments. Drug or radiation administration was adopted when the tumors reached about 50 mm^3^ in size, at which point mice were randomized for treatment with PBS (intraperitoneally), acipimox (50 mg/kg/every 2 d, intraperitoneally), 5‐FU (20 mg/kg/every 2 d, intraperitoneally), or radiation (Once, 8 Gy). For the immunotherapy group, the subcutaneous tumor model was established by inoculation of 2 × 10^6^ CT26 cells into C57BL/6 mice. Radiotherapy and drug administration was adopted when the tumors reached about 50 mm^3^ in size, at which point mice were randomized for treatment with PBS (intraperitoneally), acipimox (50 mg/kg/every 2 d, intraperitoneally), 5‐FU (20 mg/kg/every 2 d, intraperitoneally), radiation (Once, 8 Gy), or an‐PD1 antibody (200 µg/every 3 d, intraperitoneally). For org‐PDX studies, about 500 organoids were subcutaneously injected into the flanks of male BALB/C nude mice. Once the tumors reached a size of 200 mm^3^, Drug administration was adopted. Mice were euthanized by CO_2_ asphyxiation for tumor harvesting after the appearance of tumors with a diameter greater than 1.5 cm in any group. The weights of the excised tumors were recorded.

### Statistics Analysis

Statistical analysis was performed using GraphPad Prism version 8.0 for Windows. For comparing two groups, the two‐tailed Student's *t*‐test was used unless otherwise stated. All boxplots indicate median (center), 25th and 75th percentiles (bounds of box), and minimum and maximum (whiskers). Experiments were performed a minimum of three times. *P* < 0.05 was considered statistically significant. All grouped data are presented as mean ± SD unless otherwise stated.

## Conflict of Interest

The authors declare no conflict of interest.

## Author Contributions

S.Y.X., W.T.X., and L.S.R. designed and directed the study. L.H.S., Z.Z.W., X.L., L.Z.X., and L.W.X. analyzed the data, prepared the figures, and wrote the manuscript. S.Y.X., W.T.X., L.S.R., and L.W.X. performed all the experiments. K.L., H.L., Y.Y.F., and L.S.H. supervised the manuscript. H.L. provided funding for the studies.

## Supporting information



Supporting Information

Supplemental Table 1

## Data Availability

All sequencing data are available upon request from the authors. All data are available in the main text or the Supporting Information.
