# Supplementary material for: Dynamic Arginine Methylation of YBX1 Relay Controls Its Phase Separation and Chemoradiotherapy Resistance in Rectal Cancer
Source: Adv Sci (Weinh). 2025 Oct 28;12(46):e02786. doi: 10.1002/advs.202502786 (PMC12697906; doi:10.1002/advs.202502786)
Supplement: Supplementary file 1 — Supporting Information [file ADVS-12-e02786-s001.pdf]

Supporting Information

Title Dynamic arginine methylation of YBX1 relay controls its phase separation and chemoradiotherapy resistance in rectal cancer

Yunxing Shi[1,2], Taixuan Wan[1,2], Shaoru Liu[3], Huashan Liu[1,2], Ziwei Zeng[1,2], Wenxin Li[1,2], Zhenxing Liang[1,2], Li Xiong[1,2], Shuanglin Luo[1,2], Yunfei Yuan[3*], Liang Huang[1,2*], Liang Kang[1,2*]

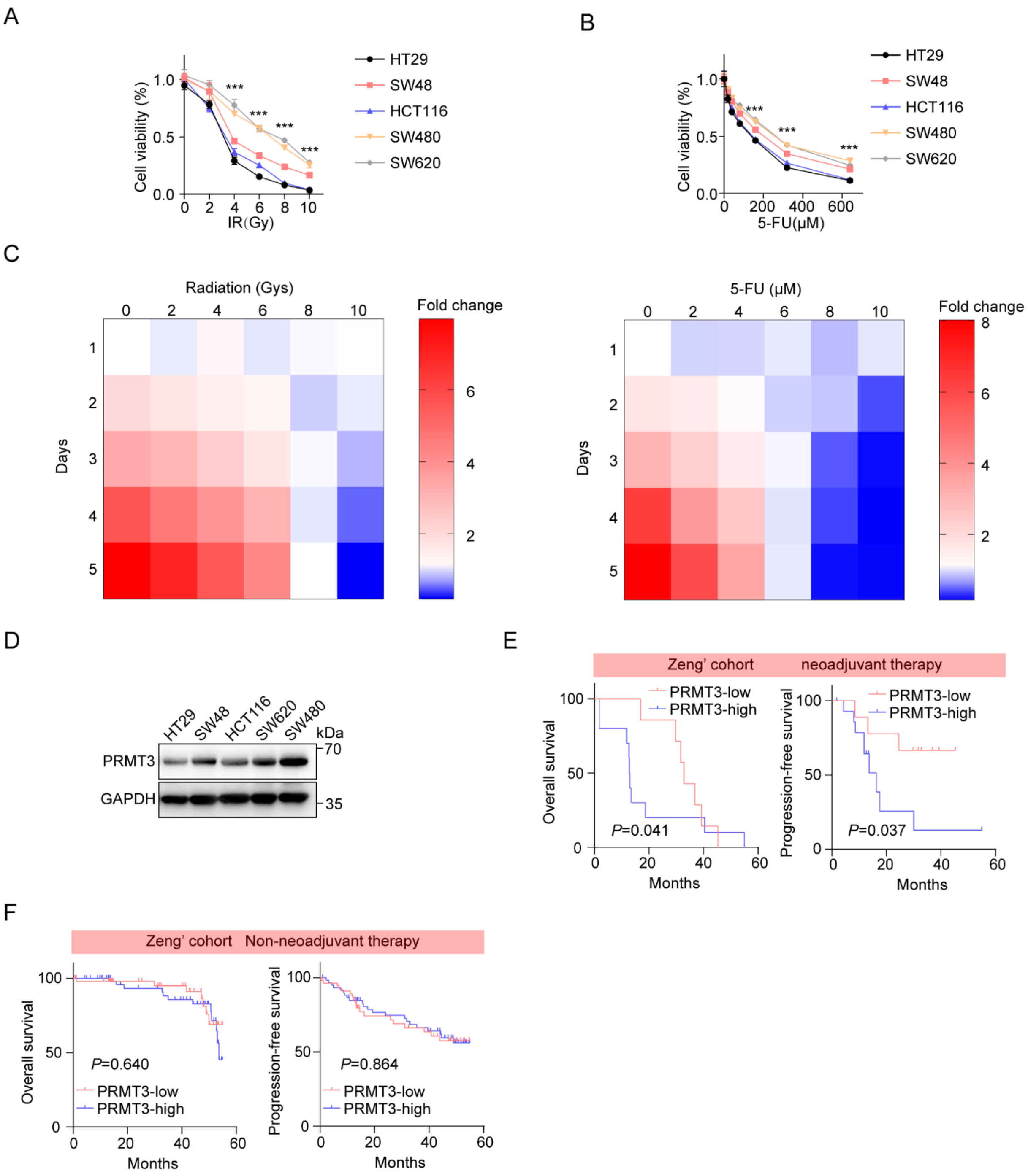

Figure S1. PRMT3 is a key driver of chemoradiotherapy resistance and associated with poor clinical outcomes

A. Dose-response curves for radiation in several CRC cell lines.

B. Dose-response curves for 5-FU in several CRC cell lines.

C. The effect of different conditions of radiation or 5-FU on proliferation inhibition ratio in SW480 cells.

D. PRMT3 expression in several CRC cell lines.

E. Kaplan–Meier overall survival and disease-free survival curves of individuals without neoadjuvant therapy with different PRMT3 expression in the public dataset cohort.

F. Kaplan–Meier overall survival and disease-free survival curves of individuals with neoadjuvant therapy with different PRMT3 expression in the public dataset cohort.

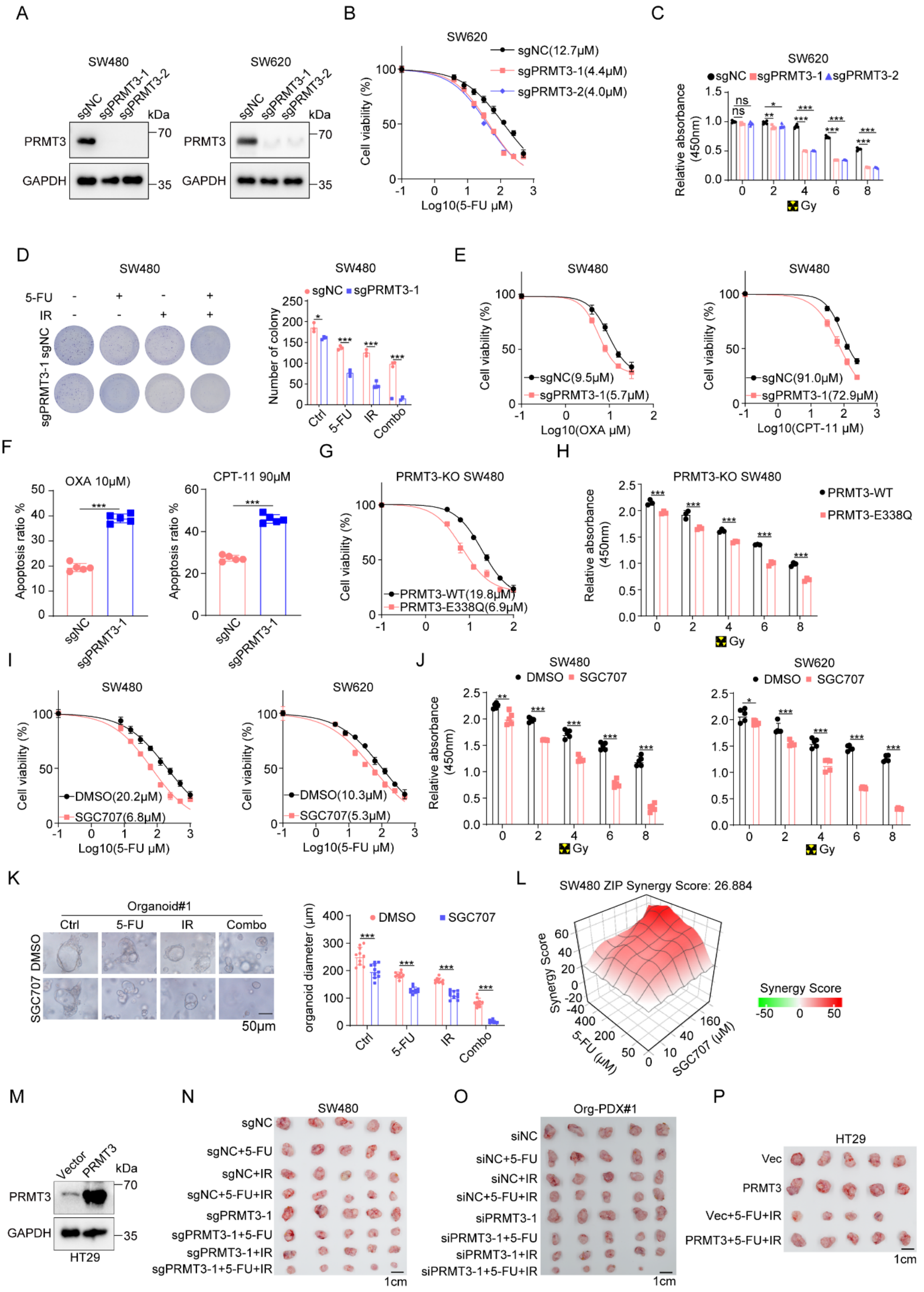

Figure S2. PRMT3 promotes chemoradiotherapy resistance in rectal cancer

A. PRMT3 expression as shown by Western blot analysis in *PRMT3*-KO cells and control cells.

B. The IC50 of 5-FU in *PRMT3*-KO and control SW620 cells.

C. Cell viability of *PRMT3*-KO and control SW620 cells after irradiation.

D. Colony number of *PRMT3*-KO and control SW620 cells after treated with 5-FU, irradiation and combination.

E. The IC50 of OXA and CPT-11 in *PRMT3*-KO and control SW620 cells.

F. Apoptosis ratio of *PRMT3*-KO and control SW480 cells after treated with OXA and CPT-11.

G. The IC50 of 5-FU in *PRMT3*-KO SW480 cells transfected with WT-PRMT3 and E338Q-PRMT3.

H. CCK8 assay to measure the effects of WT-PRMT3 and E338Q-PRMT3 on cell proliferation under irradiation treatment.

I. The effect of SGC707 (20 mM) on IC50 in SW480 and SW620 cells.

J. CCK8 assay to measure the effects of SGC707 (20 μM) on cell proliferation under irradiation treatment.

K. The effect of SGC707 (20 μM) on diameter of PDO#1 after treated with 5-FU and irradiation (4Gy).

L. Synergy score of SW480 treated with SGC707 and 5-FU.

M. PRMT3 expression as shown by Western blot analysis in *PRMT3*-OE cells and control cells.

N. The effect of *PRMT3*-KO on subcutaneously implanted SW480 cells treated with 5-FU, irradiation and combination, (n=5). Scale bar: 1cm.

O. The effect of *PRMT3*-KO on PDO#12-PDX tumor growth treated with 5-FU, irradiation and combination, (n=5). Scale bar: 1cm.

P. The effect of *PRMT3*-OE on HT29 tumor growth treated with 5-FU, irradiation and combination, (n=5). Scale bar: 1cm.

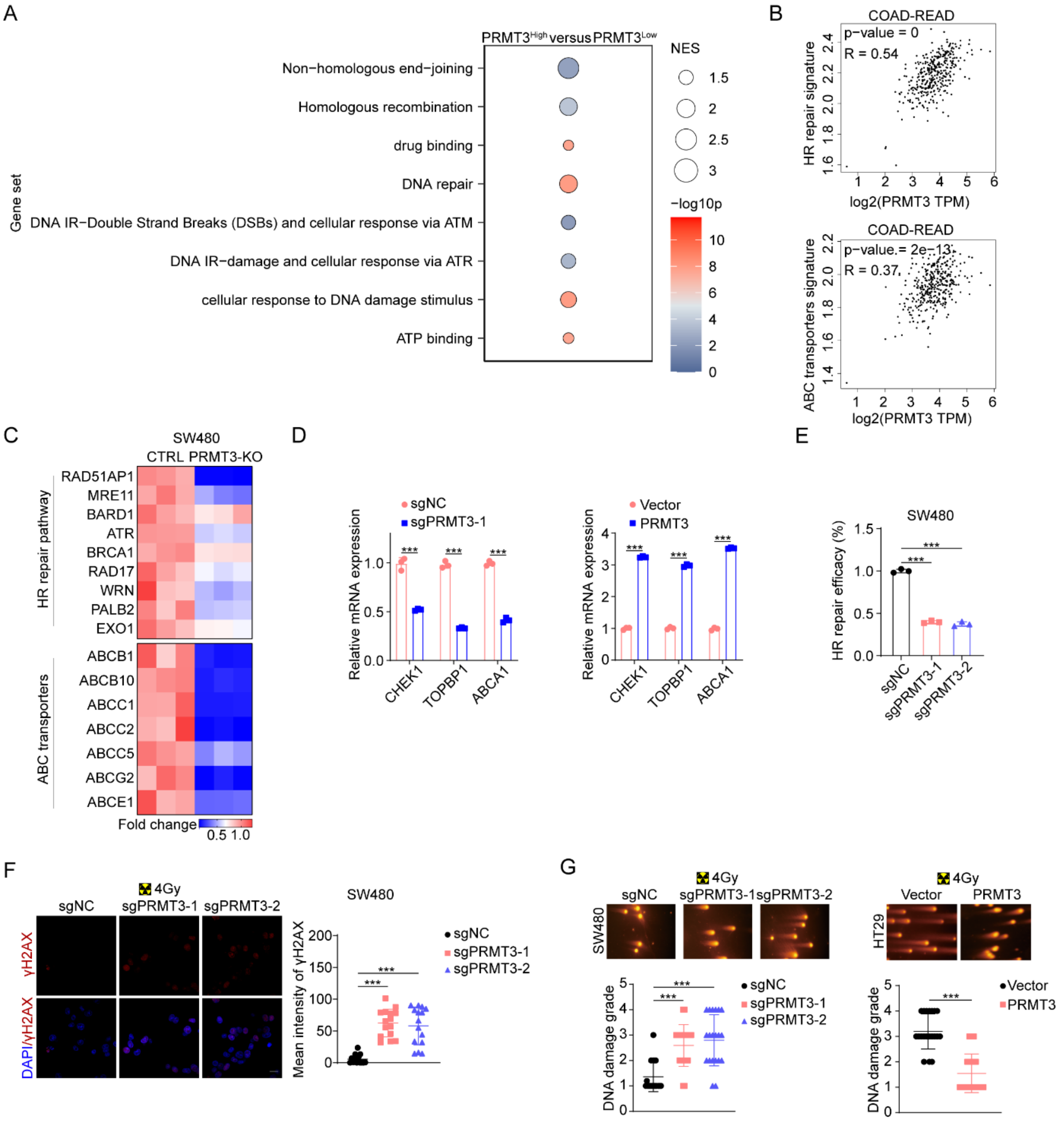

Figure S3. PRMT3 triggers homologous recombination (HR) repair and ABC transporters expression

A. GO enrichment using the gene sets containing PRMT3 upregulated and downregulated genes from TCGA-COAD/READ dataset.

B. Correlation between PRMT3 expression and HR repair signature or ABC transporters in TCGA-COAD/READ dataset.

C, D. Expression levels of HR-related genes and ABC transporters in *PRMT3*-KO versus *PRMT3*-OE SW620 cell lines, measured by qRT–PCR.

E. Quantitative analysis of HR efficiency in *PRMT3*-KO and *PRMT3*-OE SW620 cell lines.

F. Immunofluorescence staining of γH2AX in Ctrl and *PRMT3*-KO SW620 cells treated with radiation (4Gy).

G. DNA damage grade was assessed by comet assay in Ctrl and *PRMT3*-KO SW480 and *PRMT3*-OE-HT29 cells treated with radiation (4Gy).

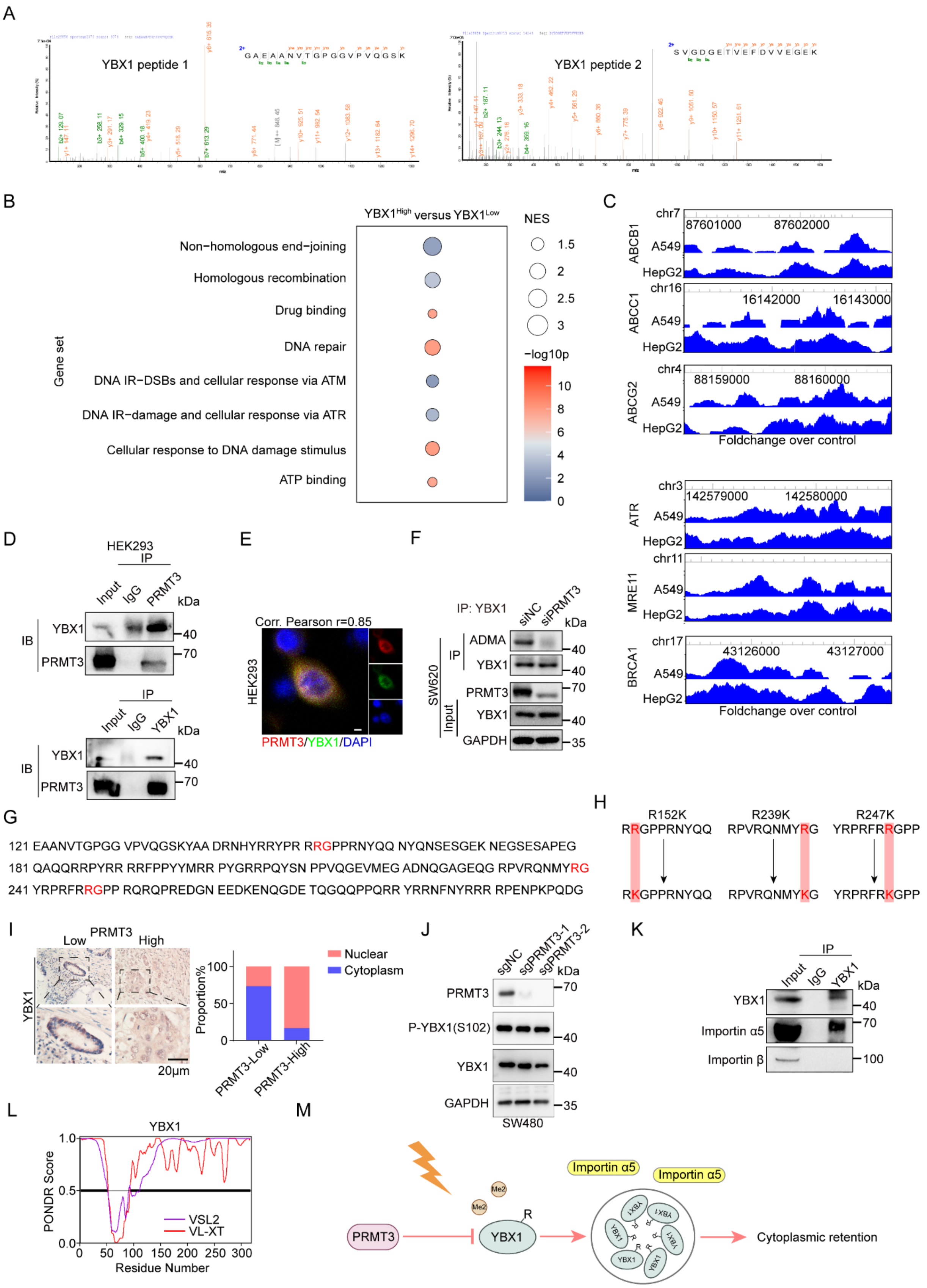

Figure S4. PRMT3 methylates YBX1 at R247 and inhibits its phase separation in

cytoplasm

A. Fragmentation spectrum of peptides identified by liquid chromatography/tandem mass spectrometry (LC-MS/MS).

B. Pathway enrichment using the gene sets containing YBX1 upregulated and downregulated genes from TCGA-COAD/READ dataset.

C. YBX1 ChIP-seq data from ENCODE shows the YBX1 binding sites at the indicated promoter region.

D. WB analysis showed that endogenous PRMT3 and YBX1 interact with each other in HEK293T cells using reciprocal co-immunoprecipitation.

E. Immunofluorescence staining showed the co-localization of PRMT3 (red) and YBX1 (green) in HEK293T cells. Scale bar, 50 mm.

F. WB analysis of immunoprecipitated YBX1 to determine the effect of *PRMT3*-KD on arginine methylation of YBX1 in SW480 cells.

G. The RG/RGG regions were indicated in the amino acid sequence of YBX1.

H. The indicated arginine was mutant to lysine.

I. IHC analysis showed YBX1 localization in CRC received neoadjuvant chemoradiotherapy.

J. WB analysis showed that *PRMT3*-KO did not influence phosphorylation of YBX1 in CRC cell line.

K. WB analysis showed that endogenous Importin α5 and YBX1 interact with each other in SW480 cells using reciprocal co-immunoprecipitation.

L. Intrinsically disordered region (IDR) prediction of YBX1.

M. Inhibiting methylation of YBX1 at R247 induces cytoplasmic phase separation, thereby

restricts YBX1's nuclear translocation in response to chemoradiation.

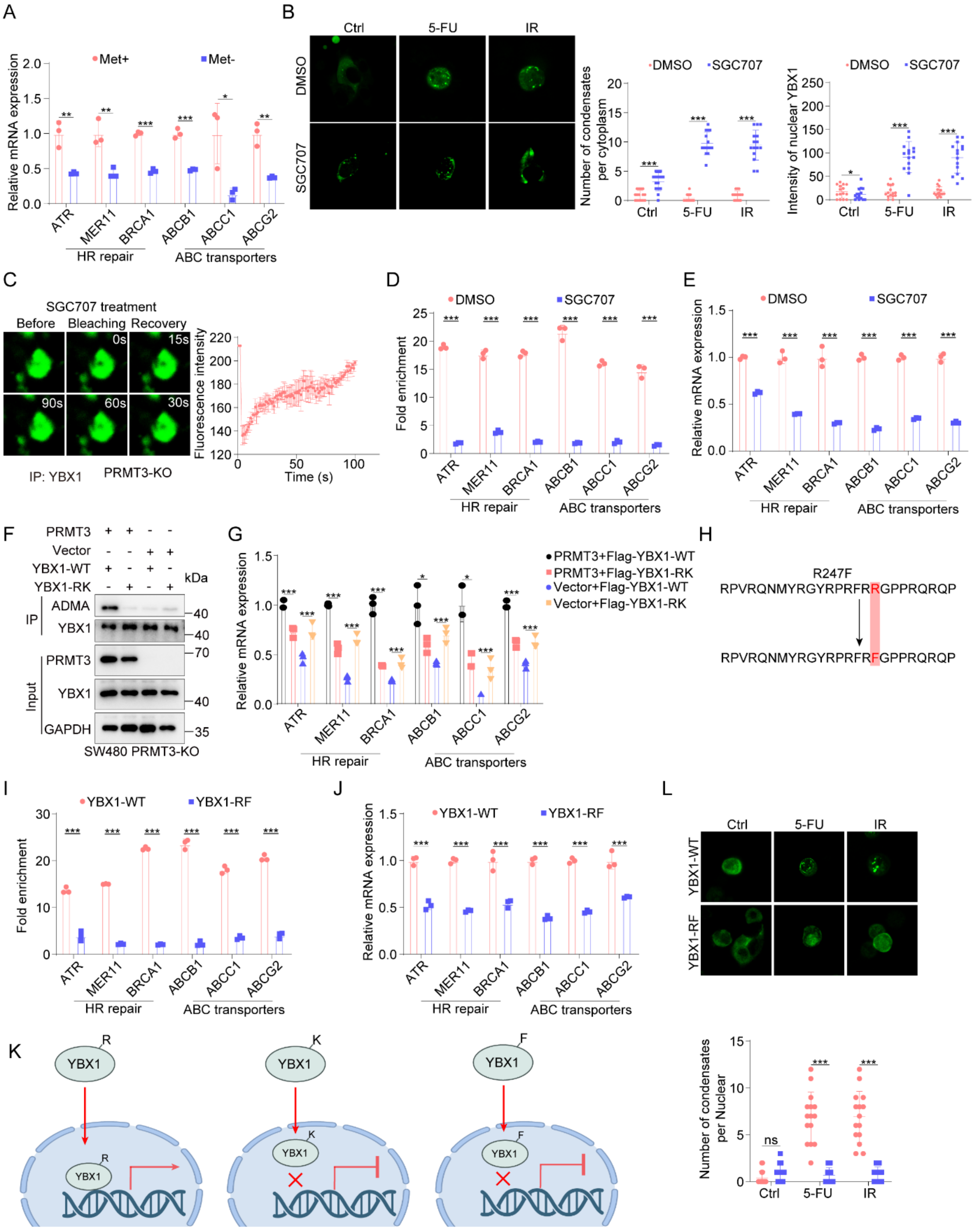

Figure S5. A methylation–demethylation of R247 relay spatiotemporally controls phase separation and transcriptive activity of YBX1

A. The differential expression of HR repair related genes and ABC transporters between CRC cells treated with Met- or Met+ culture medium.

B. Left, Live imaging of YBX1-GFP in CRC cells treated with DMSO or SGC707. Right, Quantification of intensity of nuclear YBX1 and number of condensates in cytoplasm in CRC cells treated with DMSO or SGC707.

C. B. Upper, FRAP assays in SW480 cells transfected with YBX1-GFP treated with SGC707. Lower, Quantified FRAP rate of each condensate.

D. The difference in DNA binding ability of YBX1 to its targets' promotors between SW480 cells treated with DMSO or SGC707.

E. The differential expression of HR repair related genes and ABC transporters between CRC cells treated with DMSO or SGC707.

F. WB analysis showed that PRMT3-KO SW480 cells infected with vector + YBX1-WT, vector + YBX1-RK mutant, PRMT3 + YBX1-WT, PRMT3 + YBX1-RK mutant.

G. The differential expression of HR repair related genes and ABC transporters in PRMT3-KO SW480 cells infected with vector + YBX1-WT, vector + YBX1-RK mutant, PRMT3 + YBX1-WT, PRMT3 + YBX1-RK mutant.

H. The indicated arginine was mutant to phenylalanine.

I. The difference in DNA binding ability of YBX1-WT or YBX1-R247F to its targets' promotors in SW480 cells.

J. The differential expression of HR repair related genes and ABC transporters between CRC

cells transfected with YBX1-WT or YBX1-R247F.

K. YBX1-R247K/F could not bind to promotors of targets effectively.

L. Upper, Live imaging of YBX1-WT-GFP or YBX1-R247F-GFP in CRC cells treated with chemoradiation. Lower, Quantification of number of condensates in nuclear in CRC cells.

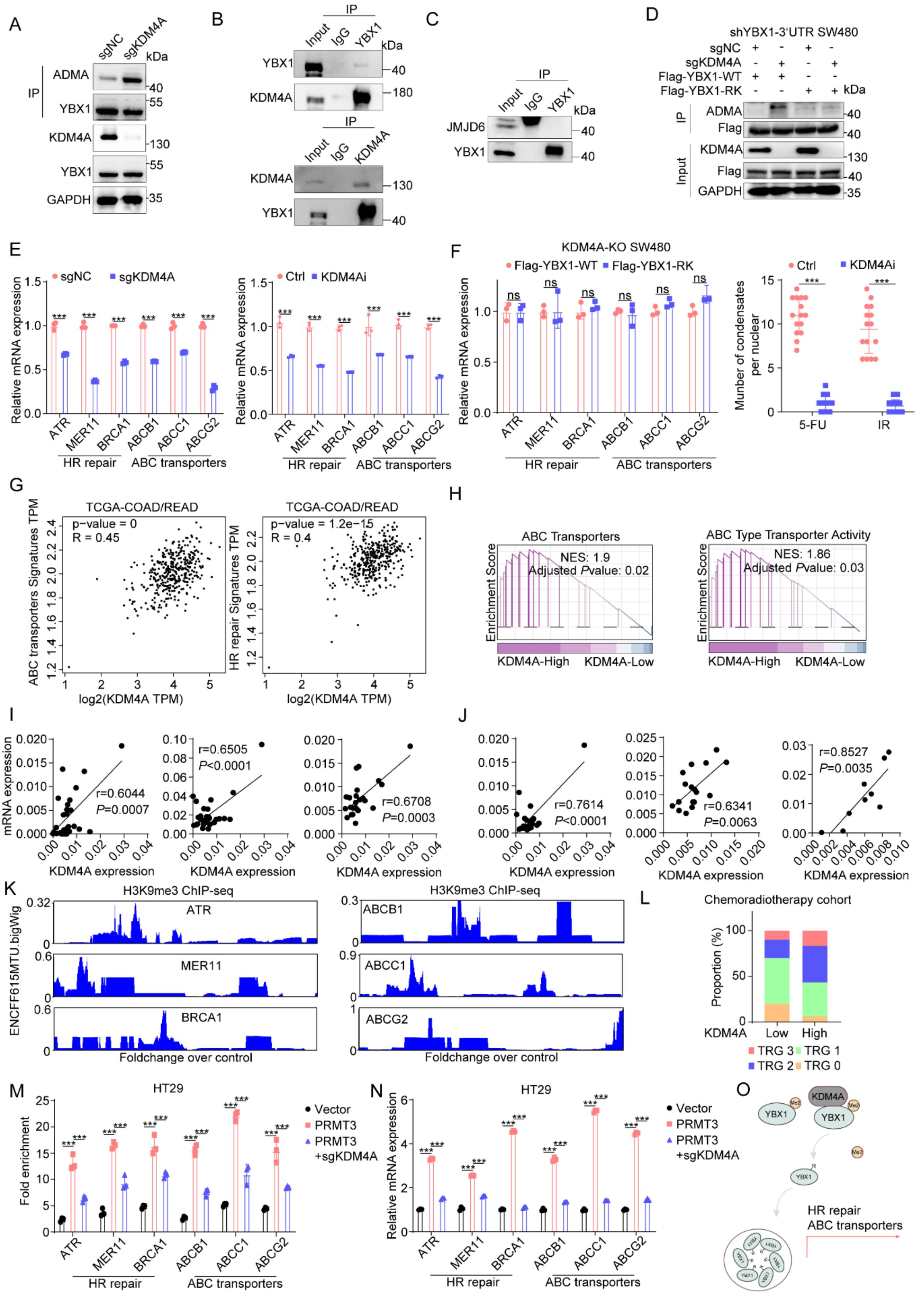

Figure S6. KDM4A demethylated YBX1 and promoted its phase separation in the nuclear.

A. WB analysis of immunoprecipitated YBX1 to determine the effect of *KDM4A*-KO on arginine methylation of YBX1 in SW480 cells.

B. WB analysis showed that endogenous KDM4A and YBX1 interact with each other in SW620 cells using reciprocal co-immunoprecipitation.

C. WB analysis showed that endogenous JMJD6 and YBX1 did not interact with each other in SW480 cells using reciprocal co-immunoprecipitation with radiation (4Gy) treatment.

D. WB analysis of Flag-YBX1-WT/R247K to determine the effect of *KDM4A*-KO on arginine methylation of Flag-YBX1-WT/R247K in SW480 cells.

E. The differential expression of HR repair related genes and ABC transporters between *KDM4A*-KO and control CRC cells or DMSO and KDM4A inhibitor treated cells.

F. The expression of HR repair related genes and ABC transporters in *KDM4A*-KO SW480 cells transfected with Flag-YBX1-WT or Flag-YBX1-R247K.

G, H. The correlation (G) and GSEA (H) analysis between KDM4A and ABC transporters and HR repair signature in TCGA-COAD/READ dataset.

I, J. The correlation between KDM4A and ABC transporters (I) and HR repair related genes (J) in CRC tissue.

K. H3K9me3 ChIP-seq data from ENCODE shows the H3K9me3 binding sites at the indicated promoter region.

L. The TRG score evaluation of rectal cancer patients, who had low and high KDM4A expression, respectively, showed the patients' response to the chemoradiotherapy.

M. The effect of *KDM4A*-KO in *PRMT3*-OE HT29 cells on the DNA binding ability of YBX1 to its targets' promotors.

N. The differential expression of HR repair related genes and ABC transporters in *PRMT3*-OE HT29 cells with or without *KDM4A*-KO.

O. KDM4A demethylates YBX1 to unmask the R247 residue and promote its phase separation in the nuclear, which strengthens its ability to transcriptionally regulate expressions of ABC transporters and HR repair genes.

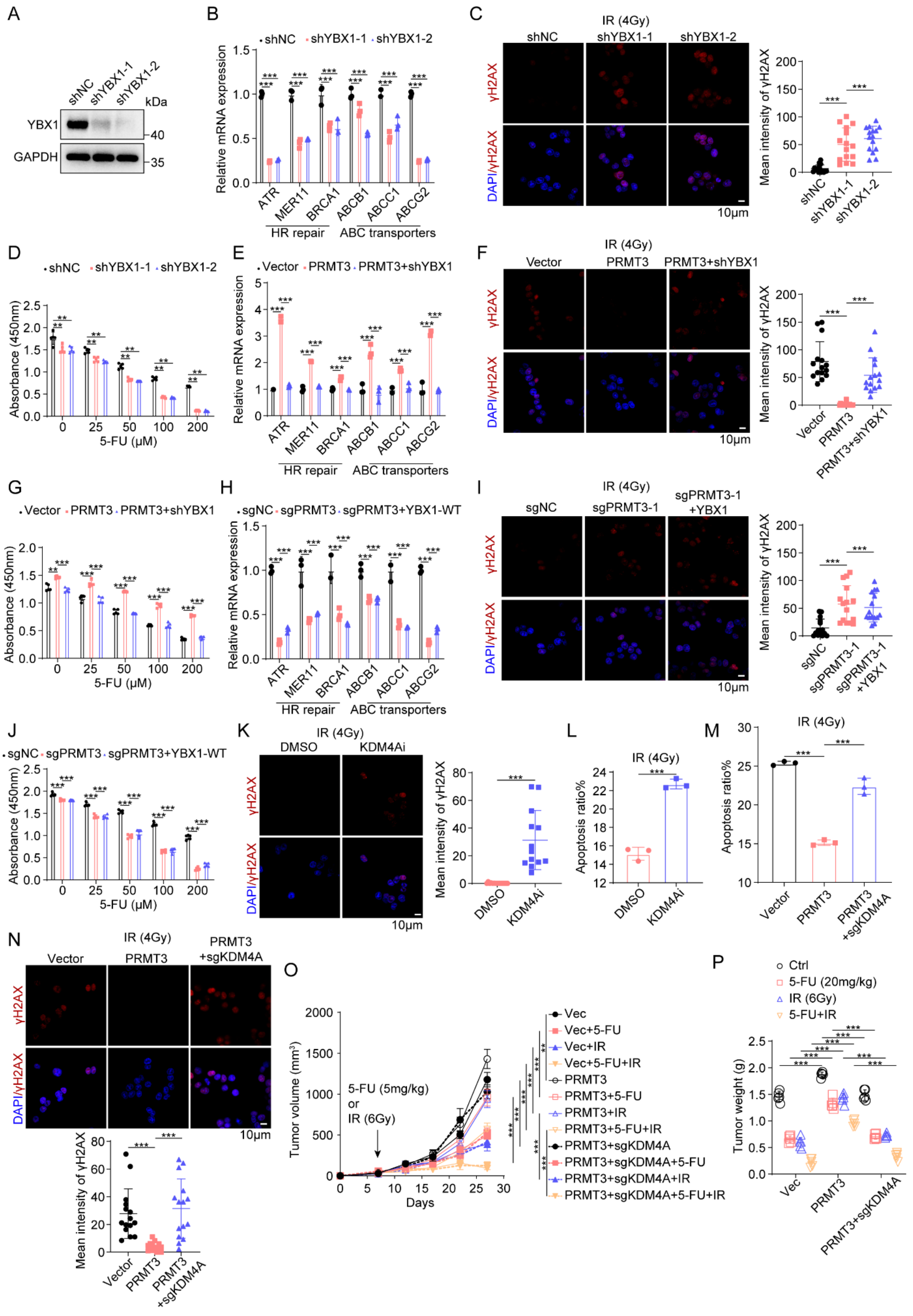
A
shNC
shYBX1-1
shYBX1-2
kDa
YBX1
40
GAPDH
35
B
shNC shYBX1-1 shYBX1-2
Relative mRNA expression
ATR MER11 BRCA1 ABCB1 ABCC1 ABCG2
HR repair
ABC transporters
C
IR (4Gy)
shNC shYBX1-1 shYBX1-2
γH2AX
DAPI/γH2AX
10μm
Mean intensity of γH2AX
D
shNC shYBX1-1 shYBX1-2
Absorbance (450nm)
5-FU (μM)
E
Vector PRMT3 PRMT3+shYBX1
Relative mRNA expression
HR repair
ABC transporters
F
IR (4Gy)
Vector PRMT3 PRMT3+shYBX1
γH2AX
DAPI/γH2AX
10μm
Mean intensity of γH2AX
G
Vector PRMT3 PRMT3+shYBX1
Absorbance (450nm)
5-FU (μM)
H
sgNC sgPRMT3 sgPRMT3+YBX1-WT
Relative mRNA expression
HR repair
ABC transporters
I
IR (4Gy)
sgNC sgPRMT3-1 sgPRMT3-1 +YBX1
γH2AX
DAPI/γH2AX
10μm
Mean intensity of γH2AX
J
sgNC sgPRMT3 sgPRMT3+YBX1-WT
Absorbance (450nm)
5-FU (μM)
K
IR (4Gy)
DMSO KDM4Ai
γH2AX
DAPI/γH2AX
10μm
Mean intensity of γH2AX
L
IR (4Gy)
Apoptosis ratio%
DMSO KDM4Ai
M
IR (4Gy)
Apoptosis ratio%
Vector PRMT3 PRMT3 +sgKDM4A
N
IR (4Gy)
Vector PRMT3 PRMT3 +sgKDM4A
γH2AX
DAPI/γH2AX
10μm
Mean intensity of γH2AX
O
5-FU (5mg/kg)
or
IR (6Gy)
Tumor volume (mm³)
Days
Vec
Vec+5-FU
Vec+IR
Vec+5-FU+IR
PRMT3
PRMT3+5-FU
PRMT3+IR
PRMT3+5-FU+IR
PRMT3+sgKDM4A
PRMT3+sgKDM4A+5-FU
PRMT3+sgKDM4A+IR
PRMT3+sgKDM4A+5-FU+IR
P
Ctrl
5-FU (20mg/kg)
IR (6Gy)
5-FU+IR
Tumor weight (g)
Vec PRMT3 PRMT3+sgKDM4A

151

Figure S7. YBX1 and KDM4A mediated the effects of PRMT3 on chemoradiation resistance.

A. YBX1 expression as shown by Western blot analysis in *YBX1*-KD SW480 cells and control cells.

B. Expression levels of HR-related genes and ABC transporters in *YBX1*-KO versus control CRC cell lines, measured by qRT–PCR.

C. Immunofluorescence staining of γH2AX in Ctrl and *YBX1*-KO SW480 cells treated with radiation (4Gy).

D. Cell viability of *YBX1*-KD and control SW480 cells with 5-FU treated.

E. The differential expression of HR repair related genes and ABC transporters in *PRMT3*-OE HT29 cells with or without *YBX1*-KD.

F. Immunofluorescence staining of γH2AX in *PRMT3*-OE HT29 cells with or without *YBX1*-KD treated with radiation (4Gy).

G. Cell viability of *PRMT3*-OE HT29 cells with or without *YBX1*-KD under 5-FU treatment.

H. The differential expression of HR repair related genes and ABC transporters in *PRMT3*-KO SW480 cells with or without *YBX1*-OE.

I. Immunofluorescence staining of γH2AX in *PRMT3*-KO SW480 cells with or without *YBX1*-OE treated with radiation (4Gy).

J. G. Cell viability of *PRMT3*-KO SW480 cells with or without *YBX1*-OE under 5-FU treatment.

K. Immunofluorescence staining of γH2AX in SW480 cells with or without KDM4A inhibitor treatment under radiation (4Gy).

L. The effect of KDM4A inhibitor on cell apoptosis of SW480 cells.

M. The effect of *KDM4A*-KO on cell apoptosis of *PRMT3*-OE HT29 cells.

N. Immunofluorescence staining of γH2AX in *PRMT3*-OE HT29 cells with or without *KDM4A*-KO treated with radiation (4Gy).

O, P. The measurement of tumor volumes (O) and tumor weight (P) to determine the effect of *KDM4A*-KO on the growth of *PRMT3*-OE HT29 cells, which were treated with 5-FU, radiation or combination (n=5).

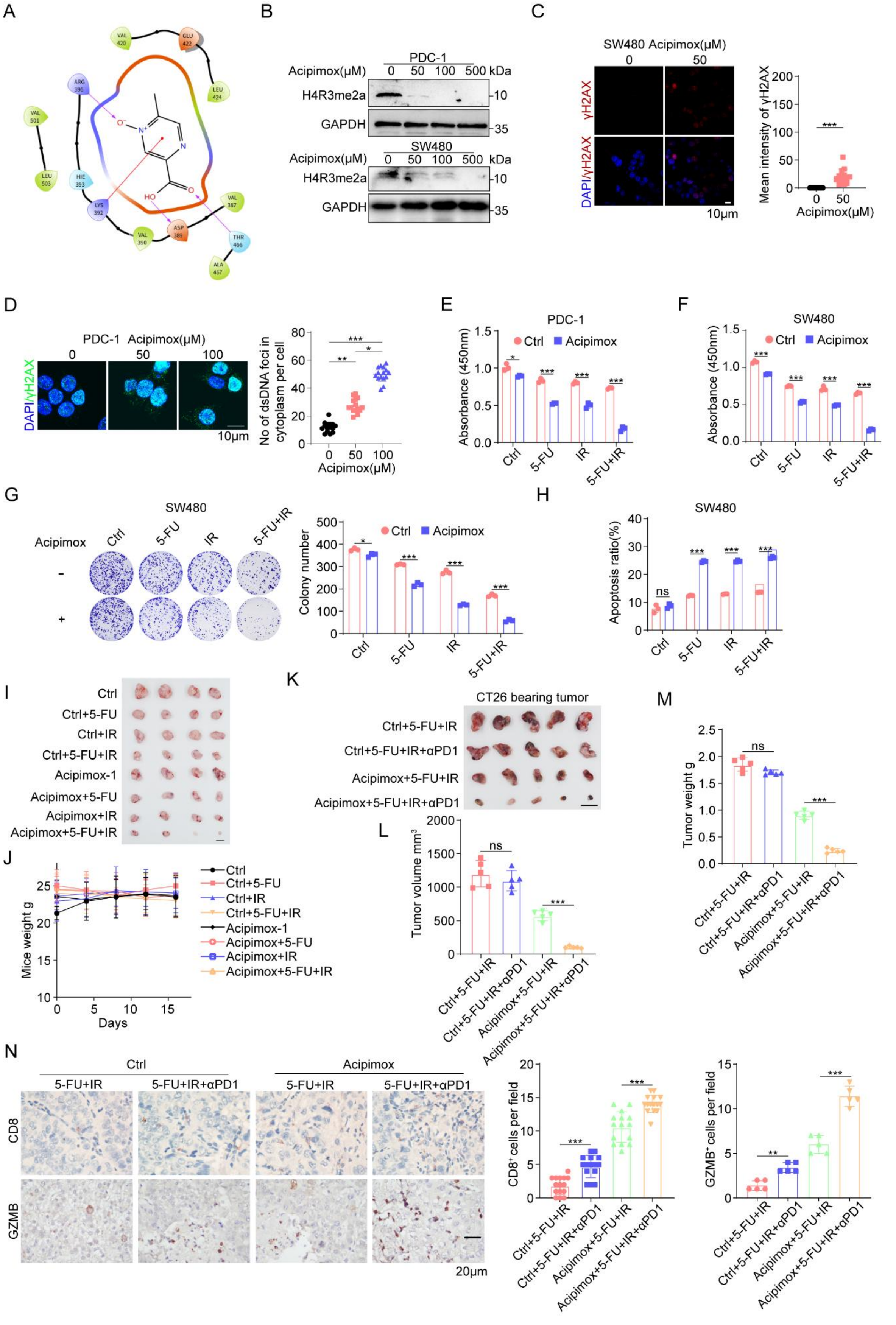
A
VAL 420
GLU 422
ARG 396
LEU 424
VAL 501
LEU 503
HIE 393
LYS 392
VAL 390
ASP 389
VAL 387
THR 466
ALA 467
B
PDC-1
Acipimox(μM) 0 50 100 500 kDa
H4R3me2a
GAPDH
-10
-35
SW480
C
SW480 Acipimox(μM)
0 50
γH2AX
DAPI/γH2AX
10μm
Mean intensity of γH2AX
Acipimox(μM)
D
PDC-1 Acipimox(μM)
0 50 100
DAPI/γH2AX
10μm
No of dsDNA foci in cytoplasm per cell
E
PDC-1
Ctrl
Acipimox
Absorbance (450nm)
Ctrl
5-FU
IR
5-FU+IR
F
SW480
G
SW480
Acipimox
Colony number
H
Apoptosis ratio(%)
ns
I
Ctrl
Ctrl+5-FU
Ctrl+IR
Ctrl+5-FU+IR
Acipimox-1
Acipimox+5-FU
Acipimox+IR
Acipimox+5-FU+IR
J
Mice weight g
Days
K
CT26 bearing tumor
Ctrl+5-FU+IR
Ctrl+5-FU+IR+αPD1
Acipimox+5-FU+IR
Acipimox+5-FU+IR+αPD1
L
Tumor volume mm³
M
Tumor weight g
N
Ctrl
Acipimox
5-FU+IR
5-FU+IR+αPD1
CD8
GZMB
20μm
CD8⁺ cells per field
GZMB⁺ cells per field

Figure S8. Acipimox allosterically inhibited enzymatic activity of PRMT3 and sensitized colorectal cancer to chemoradiation therapy

A. Computational model and interactions of acipimox and PRMT3.

B. The effect of acipimox on the H4R3me2a level in SW480 and PDC-1 cells.

C. Immunofluorescence staining of γH2AX in acipimox (50μM) treated SW480 cells under radiation (4Gy) treatment.

D. Immunofluorescence analysis of dsDNA in Ctrl and acipimox treated PDC-1 cells treated with radiation (4Gy).

E, F. Cell viability of PDC-1 (E) and SW480 (F) cells after treated with 5-FU, irradiation and combination.

G. The effect of acipimox (50μM) on colony formation ability of SW480 cells after treated with 5-FU, irradiation and combination.

H. The effect of acipimox (50μM) on apoptosis of SW480 cells after treated with 5-FU, irradiation and combination.

I. The effect of acipimox (10mg/kg) on PDO-PDX tumor growth treated with 5-FU, irradiation and combination, (n=5). Scale bar: 1cm.

J. The effect of acipimox (10mg/kg) on the mice weight during the treatment, (n=5).

K. The effect of acipimox (10mg/kg) on the response of CT26 tumor to anti-PD1 therapy on the basis of chemoradiotherapy, (n=5). Scale bar: 1cm.

L, M. The measurement of tumor volumes (L) and tumor weight (M) to determine the effect of acipimox (10mg/kg) on the response of CT26 tumor to anti-PD1 therapy on the basis of chemoradiotherapy, (n=5).

N. Representative IHC staining and quantification of $CD8^+$ T cells and $GZMB^+$ cells from indicated tumors at day 14 post-transplantation. Scale bar, 20 μm.

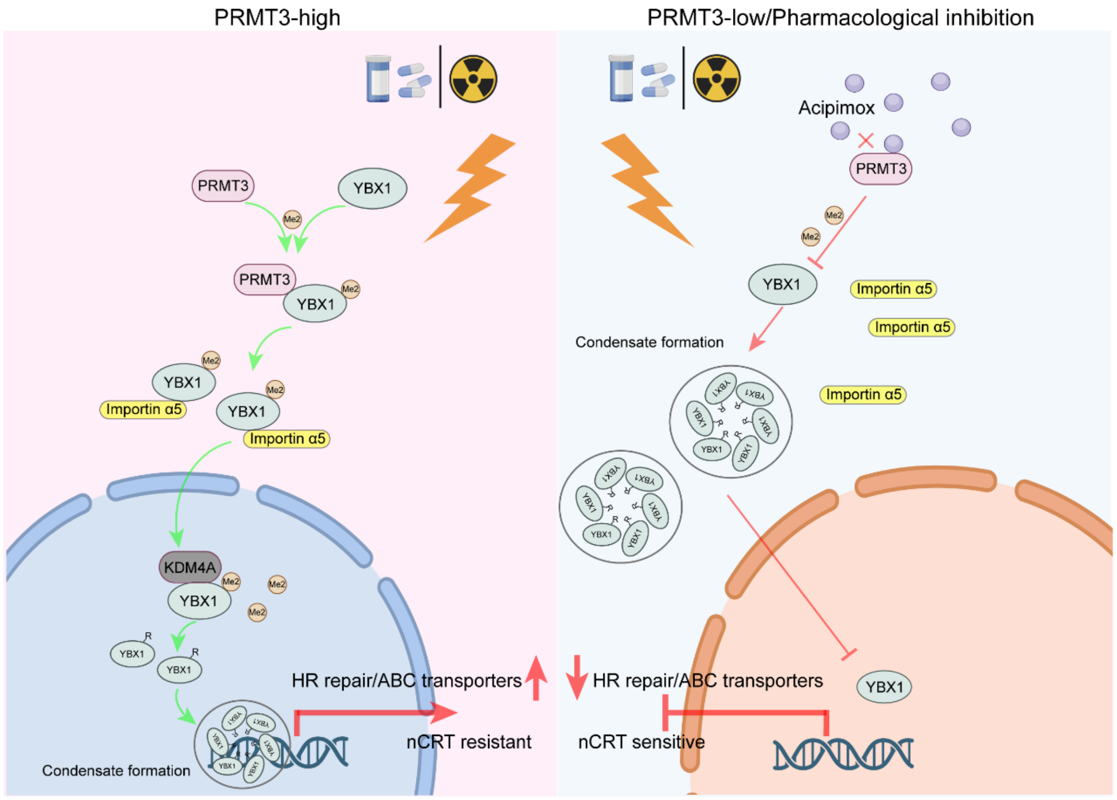

Figure S9. Working model. PRMT3 induces the arginine methylation of YBX1, which suppresses YBX1's capacity for phase separation and is imperative for its nuclear translocation. Subsequently, lysine-specific demethylase 4A (KDM4A) demethylates YBX1 to unmask the R247 residue and promote its phase separation in the nuclear, which strengthens its ability to transcriptionally regulate expressions of ABC transporters and HR repair genes and reinforces chemoradiotherapy resistance. Perturbation of either arginine methylation or demethylation sensitizes rectal cancer to chemoradiotherapy. Acipimox is a potent PRMT3 inhibitor and chemoradiotherapy sensitizer.

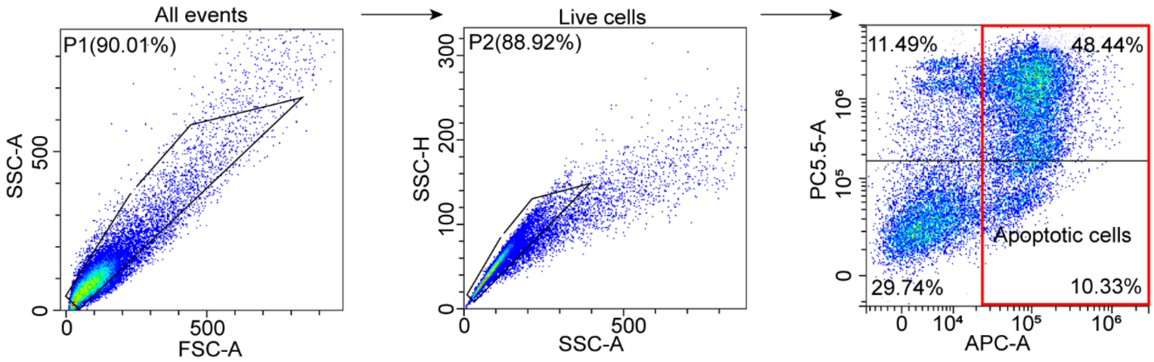

Figure S10. Gating strategy for all annexin V-APC/7-AAD apoptosis assay measured by flow cytometry.
